# Extracellular vesicles from human multipotent stromal cells protect against hearing loss after noise trauma in vivo

**DOI:** 10.1002/ctm2.262

**Published:** 2020-12-21

**Authors:** Athanasia Warnecke, Jennifer Harre, Hinrich Staecker, Nils Prenzler, Dirk Strunk, Sebastien Couillard‐Despres, Pasquale Romanelli, Julia Hollerweger, Teresa Lassacher, Daniela Auer, Karin Pachler, Georg Wietzorrek, Ulrike Köhl, Thomas Lenarz, Katharina Schallmoser, Sandra Laner‐Plamberger, Christine S. Falk, Eva Rohde, Mario Gimona

**Affiliations:** ^1^ Department of Otorhinolaryngology Head and Neck Surgery Hannover Medical School Hannover Germany; ^2^ Department of Otolaryngology, Head and Neck Surgery University of Kansas School of Medicine Kansas City Kansas; ^3^ Institute of Experimental and Clinical Cell Therapy Spinal Cord Injury and Tissue Regeneration Centre Salzburg (SCI‐TReCS) Paracelsus Medical University Salzburg Austria; ^4^ Institute of Experimental Neuroregeneration Spinal Cord Injury and Tissue Regeneration Centre Salzburg (SCI‐TReCS) Paracelsus Medical University Salzburg Austria; ^5^ Austrian Cluster for Tissue Regeneration Vienna Austria; ^6^ GMP Unit, Spinal Cord Injury and Tissue Regeneration Centre Salzburg (SCI‐TReCS) Paracelsus Medical University (PMU) Salzburg Austria; ^7^ Research Program “Nanovesicular Therapies,” Paracelsus Medical University (PMU) Salzburg Austria; ^8^ Institute of Molecular and Cellular Pharmacology Medical University of Innsbruck Innsbruck Austria; ^9^ Institute of Cellular Therapeutics Hannover Medical School and Clinical Immunology University Leipzig, Fraunhofer Institute for Cell Therapy and Immunology Leipzig Germany; ^10^ Department of Transfusion Medicine University Hospital Salzburger Landeskliniken GesmbH (SALK) and Paracelsus Medical University (PMU) Salzburg Austria; ^11^ Institute of Transplant Immunology Hannover Medical School Hannover Germany

**Keywords:** extracellular vesicles (EVs), inner ear, neuroprotection, spiral ganglion neurons, umbilical cord‐derived mesenchymal stromal cells (UC‐MSC)

## Abstract

The lack of approved anti‐inflammatory and neuroprotective therapies in otology has been acknowledged in the last decades and recent approaches are heralding a new era in the field. Extracellular vesicles (EVs) derived from human multipotent (mesenchymal) stromal cells (MSC) can be enriched in vesicular secretome fractions, which have been shown to exert effects (eg, neuroprotection and immunomodulation) of their parental cells. Hence, MSC‐derived EVs may serve as novel drug candidates for several inner ear diseases. Here, we provide first evidence of a strong neuroprotective potential of human stromal cell‐derived EVs on inner ear physiology. In vitro, MSC‐EV preparations exerted immunomodulatory activity on T cells and microglial cells. Moreover, local application of MSC‐EVs to the inner ear significantly attenuated hearing loss and protected auditory hair cells from noise‐induced trauma in vivo. Thus, EVs derived from the vesicular secretome of human MSC may represent a next‐generation biological drug that can exert protective therapeutic effects in a complex and nonregenerating organ like the inner ear.

## Graphical abstract

The Effect of extracellular vesicles derived from human stromal cells on the inner ear has been investigated. The vesicles were characterized and tested in vitro in spiral ganglion neurons and in vivo in a mouse noise trauma model.

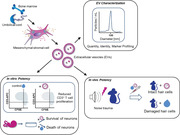

## 1 INTRODUCTION

Hearing loss is the most prevalent neurodegenerative disorder in man. One in six Europeans suffers from hearing loss,^1^ and unaddressed hearing loss produces an annual cost of over $750 billion globally.^2^ Despite being such a prevalent disorder, there is no class of inner ear drugs available up to date for treating hearing loss or associated conditions. The cochlea is the sensory part of the inner ear that is responsible for hearing. There are multiple causes of hearing loss including genetic predisposition, infections, ototoxic agents, and environmental factors such as noise and aging. Depending on the severity, different grades of damage are observed within the cochlea, which can be reversible or permanent. Loss of hair cells or auditory neurons, the main histological component of manifested sensorineural hearing loss, is irreversible. Therefore, current research focuses on the protection and regeneration of cochlear cells alongside an anti‐inflammatory/immunomodulatory treatment prior to actual cell loss. Neurotrophins such as brain‐derived neurotrophic factor (BDNF) and neurotrophin‐3 (NT‐3) regulate the connection between hair cells and auditory neurons during embryogenesis.^3^ In the adult system, hair and supporting cells within the organ of Corti release neurotrophins to stabilize the cochlear synapses.^4^, ^5^, ^6^ Indeed, restoration of synapses could be achieved after noise trauma by the induced release of NT‐3 from supporting cells.^7^ While certain protective and even immunomodulatory effects of individual neurotrophins have been observed,^8^, ^9^ a cocktail of various neuroprotective factors increased the survival of auditory neurons dramatically when compared to the effects of single factors.^10^, ^11^, ^12^ In translational approaches, clinically feasible and effective methods for the delivery of human neuroprotective factors into the spatially constricted inner ear have to be considered. Platelet‐rich plasma^13^ or autologous mononuclear cells derived from human bone marrow (BM)^14^ were investigated not only as suitable sources for regulating inflammation and mediating neuroprotective and immunomodulatory effects but also as sources of a balanced composition of various naturally occurring neuroprotective factors. Mononuclear cells and also mesenchymal progenitors within the mononuclear cells secrete significant amounts of micro‐ and nanovesicles,^15^, ^16^, ^17^, ^18^ which may contribute to neuroprotection and regulation of inflammation. These extracellular vesicles (EVs) can be enriched in a vesicular secretome fraction carrying surface markers that might differ from the marker profile found in the recipient cells. The secretome contains soluble molecules like proteins, lipids, nucleic acid species, and vesicular components such as microvesicles (100‐1000 nm), apoptotic blebs (50‐4000 nm), and small EVs (70‐150 nm), mostly referred to as “exosomes.”^19^

Exosomes were first described in the 1940s.^20^ All types of cell‐derived EVs may mediate local and systemic intercellular communication by transporting their cargo to recipient cells.^21^, ^22^ Depending on their cell of origin, EVs are involved in physiological and even pathological processes. For example, regeneration after stroke injury in rats and mice was promoted by EVs on a similar level as by transplanted cells.^23^ The pretreatment with EVs derived from mesenchymal stromal cells (MSCs) attenuated the nephrotoxic effect of cisplatin by the activation of autophagy.^24^ Depending on their cell source (eg, tumor cells, MSC‐cell type), EVs can also exert adverse effects^25^, ^26^, ^27^, ^28^ underlining the importance of strict safety evaluation of novel EV‐based therapeutics for every disease condition. In the inner ear, EVs derived from human vestibular schwannoma cells can damage cochlear hair cells.^29^ First evidence exists that EVs (derived from heat‐shocked utricles) mediate intercellular communication in the inner ear as well as protection of hair cells against neomycin‐induced hair cell death.^30^

Despite their widely expected therapeutic potential, data for only a few clinical trials testing EV‐based investigational medicinal products are available.^31^, ^32^ The results from the clinical treatments and accumulating evidence from numerous preclinical studies suggest that EVs may serve as potent and safe “cell‐derived but cell‐free” therapeutics. However, the overall safety and therapeutic effects of EV‐based biopharmaceuticals are not yet clear.

Application of MSC‐EV preparations to the inner ear has not been attempted so far. We herein show that EVs from human MSC either from umbilical cord (UC‐MSC‐EVs) or from bone marrow (BM‐MSC‐EVs) significantly improve the survival rate and neurite outgrowth of primary rat auditory neurons indicating a neuroprotective and neuroregenerative effect that is delivered across species barriers. Furthermore, treatment with MSC‐EVs can alleviate noise‐induced hair cell loss and partially restore hearing in mice in vivo even if the treatment was initiated a few days after noise trauma.

## 2 MATERIALS AND METHODS

### 2.1 Ethics

Spiral ganglion neurons (SGN) were isolated from neonatal Sprague‐Dawley rats in accordance with the German Animal Welfare Act. The euthanasia for our in vitro experiments is registered (no.: 2016/118) with the local authorities (Zentrales Tierlaboratorium, Laboratory Animal Science, Hannover Medical School, including an institutional animal care and use committee) and is reported on a regular basis as demanded by law. For exclusive sacrifice of animals for tissue analysis in research, no further approval is needed if no other treatment is applied beforehand (§4). In vivo studies were carried out on adult C57Bl/6 mice under University Kansas IACUC protocol 2018‐2442. The approval to use human MSCs for EV enrichment was obtained from the Ethics Committee of the province of Salzburg (protocol 415‐E/1776/4‐2014).

### 2.2 Primary isolation and expansion of human MSCs

Human UC‐derived MSCs were isolated as previously described.^33^ Immediately after delivery, cords were collected and stored in phosphate buffered saline (PBS) until further processing. Whole cords were washed with PBS to remove contaminating blood cells before the cord stroma was cut into small pieces of 1‐2 mm^3^. Pieces were transferred into a culture plate allowing them to dry‐adhere to the plastic surface before adding culture medium based on alpha‐modified minimum essential medium (α‐MEM, Sigma‐Aldrich) supplemented with 10% (v/v) pooled human platelet lysate (pHPL) and Dipeptiven (5.5 mg/mL, Fresenius‐Kabi, Graz, Austria). Pooled HPL was prepared as previously described,^34^ and were EV‐depleted. In brief, expired irradiated platelet concentrates were lysed by several freeze/thaw cycles. Platelet fragments were pelleted by centrifugation (4000 × *g*, 15 minutes at room temperature) and aliquots of the supernatant were frozen at −30°C until use.

**Highlights**

- Mesenchymal stromal cell‐derived extracellular vesicles (MSC‐EVs) exerted immunomodulatory activity on T cells and microglial cells.
- Spiral ganglion neuron survival was significantly improved by MSC‐EVs in vitro.
- MSC‐EVs contain brain‐derived neurotrophic factor (BDNF).
- Local application of MSC‐EVs to the inner ear attenuated hearing loss and protected auditory hair cells from noise‐induced trauma in vivo.

After 10‐12 days, outgrowing UC‐MSC colonies became visible and cord tissue pieces were removed. UC‐derived MSCs were detached enzymatically by addition of TrypLE Select CTS (A12859‐01, Gibco), and further expanded in cell factory systems (CF4, Thermo Scientific). Human bone marrow (BM)‐derived MSCs for the production of research‐grade EV preparations were purchased from AllCells (Alameda, CA). Immunophenotype and viability analysis of MSC was carried out according to the suggested marker profile for defining MSC identity as published by the International Society of Cell Therapy (ISCT) in 2005.^35^

### 2.3 Manufacturing and characterization of MSC‐EVs

We prepared independent batches of research‐grade EVs from both BM‐ and UC‐MSCs as well as clinical‐grade EV batches from human UC‐MSCs according to Good Manufacturing Practice (GMP) as previously described.^36^ In brief, cells were cultured in fibrinogen‐depleted culture medium at 5% CO_2_ and 37°C.^34^, ^36^, ^37^ Upon reaching 60‐70% confluence, growth medium was exchanged with EV‐depleted harvest medium. After 24 hours, conditioned harvest medium was centrifuged and sterile filtered (0.22 μm). Resulting supernatant was reduced and buffer‐exchanged into PBS by tangential flow filtration (TFF) and diafiltration, respectively, using a 100 kDa hollow fiber filter (Spectrum Labs). Ultimately, EVs were further enriched by ultracentrifugation at 120 000 × *g* for 3 hours at 18°C in a Sorvall model WX‐80 using a fixed angle rotor model Fiberlite F37L‐8 × 100, and the resulting pellets were resuspended in Ringer's lactate and again sterile filtered.

All clinical‐grade EV preparations were manufactured in a pharmaceutically certified class‐B clean room environment, individual doses were stored in glass vials at −80°C, and batches were tested for endotoxin levels, bacterial sterility, and the presence of mycoplasma. The presence and identity of EVs were characterized by MACSPlex surface profiling (MILTENYI, Biotec, Bergisch Gladbach, Germany) to demonstrate EV characteristics according to the established product release matrix of our manufacturing unit.^38^

### 2.4 Total protein mass determination

Total protein amounts were determined using a QuBit 3.0 Fluorometer instrument (Life Technologies) according to the manufacturer's instructions.

### 2.5 Cytokine profiling

Cytokines (IFN‐gamma, IL‐10, IL‐12p70, IL‐13, IL‐1ß, IL‐2, IL‐4, IL‐6, IL‐8, TNF‐α, ß‐NGF, and BDNF) from various preparations were analyzed using V‐Plex and U‐Plex human multiplex immunoassay kits on the MSD platform (Meso Scale Diagnostics, Rockville, MD) according to the manufacturer's instructions.

In addition, using Luminex‐based multiplex protein arrays (human 27‐Plex; M500KCAF0Y, BioRad, Hercules CA), the concentrations of SIM and epithelial and endothelial factors were determined. A miniaturized variant of the manufacturer's instructions was used.^39^ As little as 1‐2 μL of the samples was diluted with sample diluent (1:20) and incubated with multiplex beads for 45 minutes, followed by two washings steps. Afterwards, a cocktail of biotinylated secondary murine antibodies was added for 30 minutes and after final washing steps, the streptavidin‐PE was added. Greater than 50 beads per sample per analyte were detected using the BioPlex Manager 6.2 Software, and concentrations were calculated according to individual standard curves for each analyte ranging from ∼20 ng/mL to the detection limit of ∼2 pg/mL.

### 2.6 MicroRNA (miRNA) sequencing

EVs from three different UC‐MSC donors were sequenced by EXIQON (now QIAGEN) using the company's proprietary next‐generation sequencing process for microRNA and small RNA sequencing on a NextSeq 500 instrument.

### 2.7 Nanoparticle tracking analysis (NTA) in light scatter mode

To determine the size and amount of particles in the individual EV preparations, samples were analyzed in light scatter mode in a nanoparticle tracking device (ZetaView PMX 110 from Particle Metrix). Previously frozen EV preparations were used and samples were diluted to a concentration of 4‐7 × 10^7^ particles/mL in PBS. Prior to NTA analysis, the instrument was calibrated using Yellow/Green‐labeled 100 nm polystyrene standard beads (1:1 000 000 dilution in ddH_2_O). The minimum brightness was set to 20 arbitrary units (AU), temperature to 21.5°C, shutter to 70 AU, and sensitivity to 85 AU. Subsequently, data for two exposures at 11 measurement positions were acquired per sample. Based on the Stokes‐Einstein equation, particle size was calculated using the ZetaView software (PMX 110, Version 8.4.2).

### 2.8 CryoEM analysis

For each MSC‐EV sample, 4 μL was deposited on an electron microscopy (EM) grid coated with a perforated carbon film. Samples were quickly frozen by plunging in liquid ethane cooled by liquid nitrogen, using a Leica EM‐PC cryo system. EM grids were stored in cryo‐boxes maintained under liquid nitrogen, until the observation in the electron microscope. EM grids were mounted in a Gatan 626 cryo‐holder, transferred in a Tecnai F20 cryo‐electron microscope (FEI, ThermoFisher) operating at 200 kV. Images were recorded with a FEI‐Eagle camera.

### 2.9 MACSPlex surface protein profiling

The bead‐based multiplexed FACS‐based MACSPlex Exosome Kit (Miltenyi Biotec) is an assay for the analysis of surface markers present on EVs. To characterize the various MSC‐EV preparations, we used the MACSPlex kit according to the manufacturer's instructions and following a validated standard operating procedure with 5 × 10^7^ to 5 × 10^8^ total particles as input. Data acquisition was done using a FACS Canto II instrument (BD Biosciences). For additional CD73 analyses, an anti‐CD73‐BV421 antibody (BD Biosciences) was used. Data normalization was directed toward CD9/CD63/CD81 APC signal. Isotype control normalization was performed as described earlier.^38^, ^40^

### 2.10 Assessment of T‐cell growth inhibition potential of EV preparations in vitro

To investigate the immunomodulatory activity of clinical‐grade and research‐grade EV preparations, we analyzed the capacity to inhibit T‐cell proliferation in vitro, as described previously.^41^ Briefly, carboxyfluorescein succinimidyl ester (CFSE) prelabeled pooled peripheral blood mononuclear cells were stimulated with the mitogen phytohemagglutinin (PHA) and cocultured with different ratios of EVs for 4 days. The percentage of inhibition of fluorescently‐labeled CD3 T‐cell proliferation was analyzed by flow cytometry; data are presented in representative original dot plots and as mean ± standard deviation after measurements in triplicates. For normalization, the standard stimulation (PHA only, left dot plot, upper left quadrant) was assigned to a value of 100%, and the percentage of inhibition was calculated with 10 000 CD3+ T cells gated per analysis.

### 2.11 Analysis of anti‐inflammatory potential of EV preparations in microglial cell line BV‐2

The BV‐2 microglial cell line^42^ was maintained in Dulbecco's Modified Eagle's Medium (Merck Millipore) containing 2.2 g/L glucose, supplemented with 10% fetal bovine serum (Gibco, Cat 10270106; Lot 42F0052K), 100 U/mL penicillin and 100 μg/mL streptomycin (Pan Biotech) at 37°C under 5% CO_2_ culture conditions. To activate microglia cells, subconfluent cultures of BV‐2 cells were treated for 2 hours or 24 hours with 100 ng/mL lipopolysaccharide (LPS; Sigma‐Aldrich, Cat L6529, Lot 126M4087V, 2 100 000 EU/mg), or with PBS as control. The impact of MSC‐EVs on microglial activation was scrutinized by applying 1.2 × 10^8^ particles/mL in the culture dish, 1 hour before LPS treatment.

For the analysis of NF‐κβ p65 phosphorylation, BV‐2 cells were seeded on poly‐d‐lysine‐coated coverslips (5 μg/mL) (Millipore Cat A‐003‐E, Lot #90124‐1). Two hours after LPS application, cells were fixed for 30 minutes with 0.1 M phosphate‐buffered 4% paraformaldehyde, pH 7.4. Immunodetection was performed as described previously^43^ using a rabbit anti‐phospho‐NF‐κβ p65 (Ser536) (clone 93H1, Cell Signaling, Cat 3033, 1:2500) primary antibody, followed by donkey anti‐rabbit Alexa 488 conjugated secondary antibody (Invitrogen Cat A21206, 1:2000). Nuclear counterstain was obtained with DAPI (0.5 mg/mL) prior to mounting using Prolong Gold Antifade mounting media (Invitrogen Cat P36390). Densitometric analysis of cytoplasmic phospho‐NF‐κβ p65 staining intensity in the presence or absence of MSC‐EVs at the same concentration of 1.2 × 10^8^ particles/mL was performed using ImageJ^44^ on cells (n > 110) present in six randomly selected fields of view for two technical replicates for each experimental condition.

For analysis of gene expression, total RNA from BV‐2 cells was isolated using RNeasy Mini Kit (Qiagen) according to the manufacturer's protocol 24 hours after LPS application. Total RNA concentrations were determined with a NanoVue plus (GE Healthcare, UK). RNA was reverse transcribed into first‐strand cDNA using the iScript TM reverse transcription supermix for RT‐qPCR (Bio‐Rad Laboratories, CA) according to the manufacturer's protocol. Quantitative gene expression analyses were performed using TaqMan RT‐PCR technology. Technical duplicates containing 10 ng of reverse‐transcribed RNA were amplified with the GoTAQ Probe qPCR Master Mix (Promega) using a two‐step cycling protocol (95°C for 15 seconds, 60°C for 60 seconds; 40 cycles, Bio‐Rad CFX 96 Cycler). The following validated exon‐spanning gene expression assays were employed: Heatr3 Mm.PT.56.8463165; PSMD4 Mm.PT.56.13046188; IL‐1ß Mm.PT.56a.4161645; IL‐6 Mm.PT.56a.10005566; TNFα Mm00443258_m1, and TGF‐ß Mm.PT.56a.11254750 from Integrated DNA Technologies. The relative expression levels of the target genes were normalized on two validated housekeeping genes, Heatr3 and PSMD4.^45^ Cq values were analyzed using qBasePlus v. 2.4 (Biogazelle NV, Zwijnaarde, Belgium). Expression of target genes in control and treatment conditions were normalized to represent the relative expression in terms of “fold changes.”

### 2.12 Primary rat SGN cell culture

Neonatal (postnatal days 3‐5) Sprague‐Dawley rats of both sexes were used for preparing the primary SGN cell culture. Isolated cochleae were microscopically dissected, followed by enzymatic and mechanical dissociation of the spiral ganglia, which was performed according to a previously described protocol.^46^ The dissociated SGN cell culture consists of mixed cell types such as neurons, fibroblasts, and glial cells. Viable cells were counted by trypan blue exclusion using a Neubauer chamber. Before cell seeding, plates were coated with poly d/l‐ornithine (0.1 mg/mL; Sigma‐Aldrich) and laminin (0.01 mg/mL; natural from mouse, Life Technologies, Carlsbad, CA), as described in detail previously.^46^ The dissociated cells were seeded at a density of 1 × 10^4^ cells per well in 96‐well plates (TPP, Switzerland). The SGN were either cultivated in SGN medium only (medium, negative control), in a 1:1 mixture of SGN medium with Ringer's lactate (ringer, negative control), in the presence of 50 ng/mL BDNF (BDNF, positive control) or with escalating doses of EVs derived from 1‐4 × 10^6^ UC‐MSC or BM‐MSC. Additionally, the SGN were treated with mock EVs (produced from nonconditioned MSC medium subjected to TFF and ultracentrifugation). The SGN medium consisted of Panserin 401 (PAN Biotech, Aidenbach, Germany) supplemented with HEPES buffer (23.43 mM; Invitrogen), PBS (0.172 mg/mL; PBS tablets, Gibco by Life Technologies), glucose (0.15%; B. Braun, Melsungen, Germany), penicillin (30 U/mL; Biochrom, Germany), N2‐supplement (0.1 μg/mL; Invitrogen), and insulin (8.7 μg/mL; Biochrom, Germany). After 48 hours at 37°C and 5% CO_2_, the cells were fixed with a 1:1 acetone (J. T. Baker, Deventer, Netherlands)/methanol (Roth, Karlsruhe, Germany) solution for 10 minutes and were washed with PBS. A seeding control was already fixed 4 hours after the seeding of SGN.

### 2.13 Immunostaining, survival rate, neurite length, and morphology of SGN

For identification of SGN within the heterogeneous mixture of neurons, fibroblasts, and glial cells, a neuron‐specific staining with a mouse 200 kD neurofilament antibody (clone RT97; Leica Biosystems, Wetzlar, Germany) was performed. As previously described, the Vectastain Elite ABC Kit was used according to the manufacturer's instructions.^47^ Afterwards, diaminobenzidine was added for visualization (Peroxidase Substrate Kit DAB; Vector Laboratories Inc., Burlingame, CA). Surviving neurons were defined as neurofilament‐positive cells with a neurite length of at least three cell soma diameters,^48^ and were counted by using an inverted light microscope (Olympus CKX41, Hamburg, Germany). The neuroprotective effect was determined by relating the number of survived SGN after 48 hours to the mean number of neurons in the seeding control after 4 hours of the same plate.

To examine the neurite length and a potential regenerative effect of MSC‐EVs, the five longest neurons in each field of view (one in the center and four around the perimeter of the well) were imaged using the inverted light microscope with a CCD camera (Colorview III, SIS, Olympus). Finally, the neurites were measured by using the polygon function of the imaging software CellSense Dimension (Olympus). The conditions were blinded for the analyst.

For analysis of the neuronal morphology, SGN were counted and classified into five groups: monopolar, bipolar, multipolar, pseudomonopolar neurons, and neurons with no neurites (according to Whitlon et al, 2007^49^ and Schwieger et al, 2015^12^). Neurons, which could not be clearly identified and neurons in clumps were not considered. The percentages of the five different morphologies were calculated in relation to the total number of counted neurons per well.

Statistical analysis was performed with Graph Pad Prism 5 and 7 (GraphPad, La Jolla, CA). The data were validated by using one‐way ANOVA followed by Bonferroni's multiple comparison test (morphology) and Dunnett's multiple comparison test (survival rates). *P*‐values of less than .05 were considered statistically significant. Quantitative data are presented as mean ± standard error of the mean. Levels of significance are indicated as: *P < .05; **P < .01; ***P < .001.

### 2.14 In vivo evaluation of the effects of MSC‐EVs in a noise‐induced trauma model in mice

#### 2.14.1 Animals

One‐month‐old female C57BL/6 mice (Jackson Laboratories) weighing between 18 and 23 g were utilized for all experiments. All procedures were performed under anesthesia consisting of a mixture of ketamine (100 mg/kg) and xylazine (10 mg/kg) via intraperitoneal injection.

#### 2.14.2 Evaluation of hearing

Animals were anesthetized and then placed inside a double‐walled, sound‐attenuated chamber. Body temperature was maintained at 37°C using a MediHeat V500Vstat heated operating table digital thermostat (PECO Services). Hearing thresholds were determined by auditory brainstem response (ABR) measurements using the Smart EP program from Intelligent Hearing Systems (IHS, Miami, FL). Needle electrodes were placed on the vertex (+), behind the left ear (−), and behind the opposite ear (ground). Tone bursts were presented at 4, 8, 16, and 32 kHz, with a duration of 500 microseconds using a high‐frequency transducer. Recording was carried out using a total gain equal to 100 K and using 100 Hz and 15 kHz settings for the high‐ and low‐pass filters. A minimum of 128 sweeps was presented at 90 dB sound pressure level (SPL). The SPL was decreased in 10 dB steps. Near the threshold level, 5 dB SPL steps using up to 1024 presentations were carried out at each frequency. Threshold was defined as the SPL at which at least one of the waves could be identified in two or more repetitions of the recording. Outer hair cell function in response to EV delivery was evaluated by distortion product otoacoustic emission (DPOAE) testing. Using the IHS DPOAE program, distortion products were measured for pure tones from 2 to 32 kHz using the IHS high‐frequency transducer. The Etymotic 10B+ Probe was inserted to the external ear canal. L1 level was set to 65 dB and L2 level was set to 55 dB. Frequencies were acquired with a F2‐F1 ratio of 1.22 using 16 sweeps. Pre‐ and posttreatment hearing tests were compared by ANOVA for repeated measures with a Tukey post hoc test (Prism V8).

#### 2.14.3 Evaluation of the effect of MSC‐EVs on normal hearing

To determine if MSC‐EV preparations had any toxic effects on the inner ear, normal‐hearing mice were treated with MSC‐EVs. Prior to delivery, all animals underwent evaluation of baseline hearing with ABR to check thresholds and DPOAE to evaluate outer hair cell function. For the delivery of EVs, mice were anesthetized. A dorsal postauricular incision was made, and the posterior semicircular canal exposed. Using a microdrill, a canalostomy was created, exposing the perilymphatic space. Subsequently, 1 μL of MSC‐EVs was injected using a Hamilton microsyringe with 0.1 μL graduations and a 36‐gauge needle. The canalostomy was sealed with bone wax. Five days after the delivery of MSC‐EVs, mice were again anesthetized and hearing was evaluated with ABR and DPOAE.

#### 2.14.4 Effect of MSC‐EVs on hearing in a noise trauma mouse model

Pretreatment hearing thresholds were measured by ABR 72 hours prior to the first sound exposure, and the final postoperative threshold was measured before sacrificing the animals 4 weeks after noise trauma. For the noise‐trauma (sound exposure), the mice were anesthetized as described above. Mice were then exposed to a 16 kHz pure tone presented at 118 dB SPL in the left ear for 3 (Group 1) or 2 (Group 2) hours. Sound was delivered through a speaker equipped with a ribbon tweeter (Radio Shack 40‐1310 Horn Super Tweeter). The speaker was coupled to the left ear via a short plastic tube, 12 mm in inner diameter and 45 mm in length. Prior to exposure, the sound was calibrated using a Quest Electronics Precision Integrating Sound Level Meter (model 1800). The Sound Level Meter was calibrated using a 1000 Hz Bruel & Kjaer 4230 Sound Level Calibrator. After sound exposure, animals were allowed to recover for 72 hours prior to delivery of the EVs. 1 μL of EVs derived from UC‐MSC (n = 5) or 1 μL artificial perilymph as control (n = 5) were delivered. Hearing was retested by ABR 30 days post noise trauma, and subsequently animals were processed for histology.

#### 2.14.5 Histology and immunocytochemistry of murine cochleae

Mice were anesthetized with intraperitoneal applications of phenobarbital (585 mg/kg) and phenytoin sodium (75 mg/kg) (Beuthanasia‐D Special, Schering‐Plough Animal Health Corp., Union, NJ, Canada), and sacrificed via intracardiac perfusion with 4% paraformaldehyde in PBS. The temporal bones were removed, the stapes extracted and the round window was opened. The temporal bones were postfixed overnight in 4% paraformaldehyde in PBS at 4°C. After rinsing in PBS three times for 30 minutes, the temporal bones were decalcified in 10% ethylene diamine tetracetic acid for 48 hours and embedded in paraffin.

The 7 μm sections were cut in parallel to the modiolus, mounted on Fisherbrand Superfrost/Plus Microscope Slides (Fisher Scientific, Pittsburgh, PA) and dried overnight. Samples were deparaffinized and rehydrated in PBS two times for 5 minutes, then three times in 0.2% Triton X‐100 in PBS for 5 minutes and finally in blocking solution 0.2% Triton X‐100 in PBS with 10% fetal bovine serum for 30 minutes at room temperature. Specimens were treated with antimyosin VIIa rabbit polyclonal antibody (Proteus BioSciences, Inc., Ramona, CA) diluted 1:100 in blocking solution. The tissue was incubated for 48 hours at 4°C in a humid chamber. After three rinses in 0.2% Triton X‐100 in PBS, immunohistochemical detection was carried out with rabbit‐specific HRP/DAB (ABC) Detection IHC Kit (Abcam, ab64261). The secondary antibody was incubated for 6 hours at room temperature in a humid chamber. The slides were rinsed in 0.2% Triton X‐100 in PBS three times for 5 minutes and finally coverslipped with ProLong Gold antifade reagent (Invitrogen Molecular Probes, Eugene, OR).

## 3 RESULTS

### 3.1 Characterization of MSCs and their EVs

**FIGURE 1:**
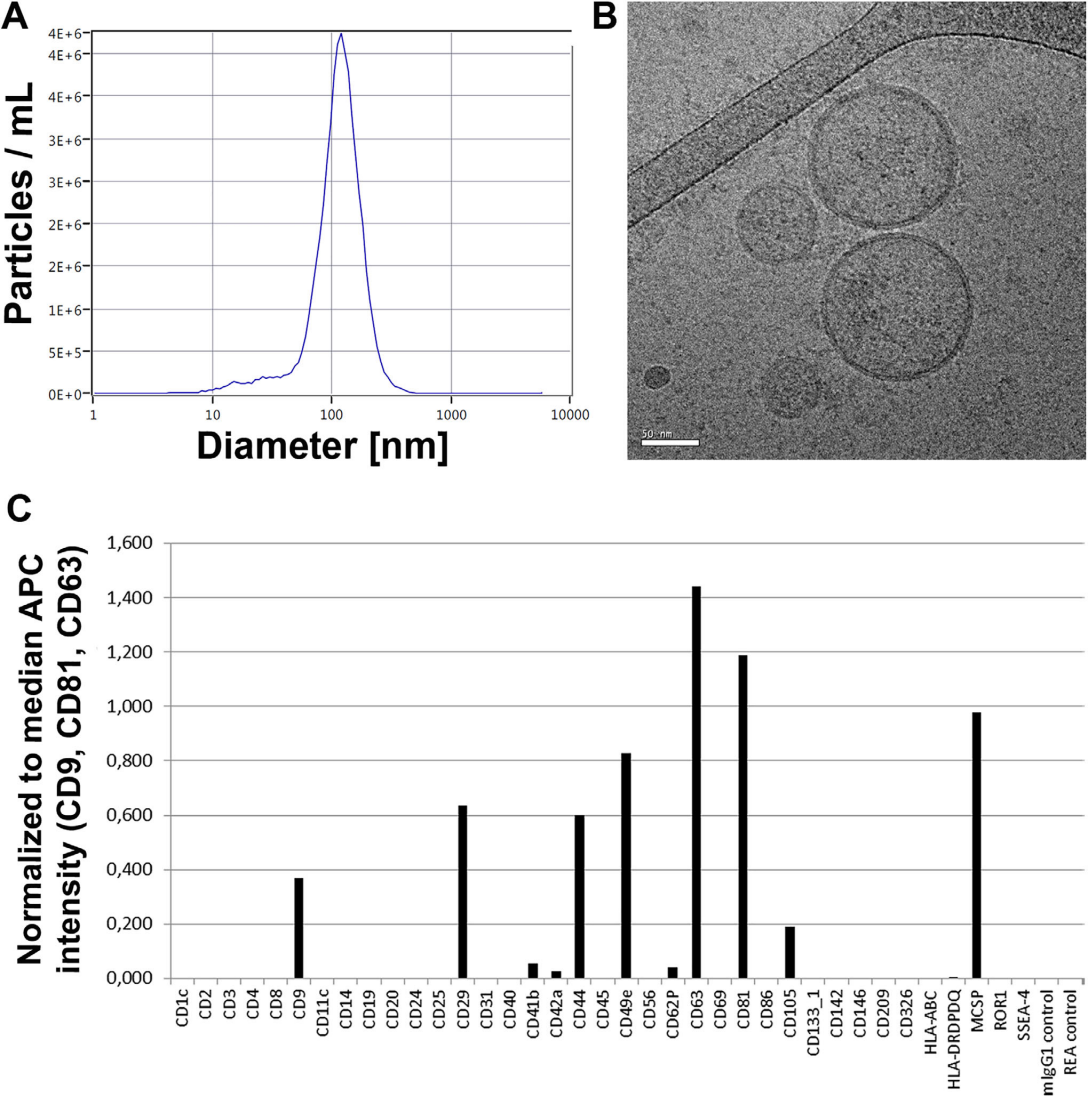
Characterization of mesenchymal stromal cell‐derived extracellular vesicles (MSC‐EVs). A, Nanoparticle tracking analysis (NTA, light scatter mode) reveals the size distribution of particles within the umbilical cord (UC)‐derived MSC‐EVs with a mean particle diameter of 110‐130 nm. B, Cryo transmission electron microscopic image of a representative UC‐MSC‐EV preparation shows double‐layer lipid membranes around spherical objects characteristic for extracellular vesicles (EVs, scale bar: 50 nm). C, Surface profiling of UC‐MSC‐EVs by MACSplex multiplex assay confirms the presence of tetraspanins (CD9, CD63, CD81) typical for EVs in addition to CD29 (Integrin beta‐1), CD44 (receptor for hyaluronic acid), CD49e (integrin alpha‐5), and melanoma‐associated chondroitin sulfate proteoglycan (MCSP)

MSCs were immunophenotyped by flow cytometry at the time of collection of the conditioned medium to determine surface marker profile of the secreting cells. All BM‐ and UC‐MSCs displayed typical MSC marker profiles (Table S1 and Figure S1). Moreover, the trilineage differentiation capacity of the used MSCs was demonstrated (Figure S2). NTA of EV preparations revealed a mean particle diameter in the range of 110‐130 nm (Figure 1A). Cryo transmission EM identified round objects of around 100 nm surrounded by a lipid bilayer, indicative of EVs (Figure 1B). Surface profiling demonstrated the presence of EV markers CD9, CD63, and CD81 in addition to specific MSC‐EV markers CD29, CD44, and CD49e (Figure 1C) and CD73 (Figure S3).

Total protein mass of EV preparations ranged between 1.7 and 4.6 mg/mL. Profiling of miRNAs by next‐generation sequencing identified three targets (hsa‐miR‐146a‐5p, hsa‐miR‐148a‐3p, and hsa‐miR‐21‐5p) among the top seven results in three individual clinical‐grade MSC‐EV samples (Table S2).

**FIGURE 2:**
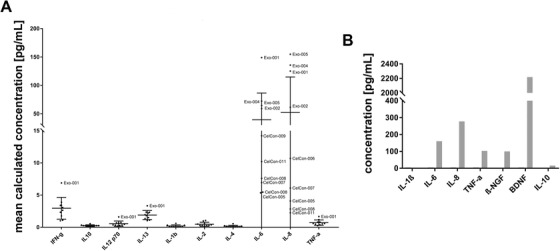
Cytokine profile of mesenchymal stromal cell‐derived extracellular vesicles (MSC‐EVs). A, Cytokine profiling for proinflammatory cytokines in multiple clinical‐grade (CelCon) and research‐grade (Exo) EV batches from umbilical cord (UC)‐derived MSC isolated by tangential flow filtration (TFF) and subsequent ultracentrifugation is shown. B, Cytokine profiling including brain‐derived neurotrophic factor (BDNF) for one representative EV preparation is shown

The presence of proinflammatory cytokines was monitored by a multiplex assay. While moderate levels of IL‐6 (ranging from 5 to 180 pg/mL) and IL‐8 (from 2 to 300 pg/mL) were detected in various research‐ and clinical‐grade EV preparations, the profile of other proinflammatory cytokines (IFN‐gamma, IL‐10, IL‐12p70, IL‐13, IL‐1ß, IL‐2, and TNF‐α) including BDNF displayed very low levels (Figure 2). Additional cytokines, chemokines, and tissue factors were quantified using a multiplex protein array. A list with all measured proteins is shown in Figure S4.

### 3.2 Reduced T‐cell proliferation as well as a lower proinflammatory potential in a microglial cell line in presence of EVs derived from UC‐MSCs

**FIGURE 3:**
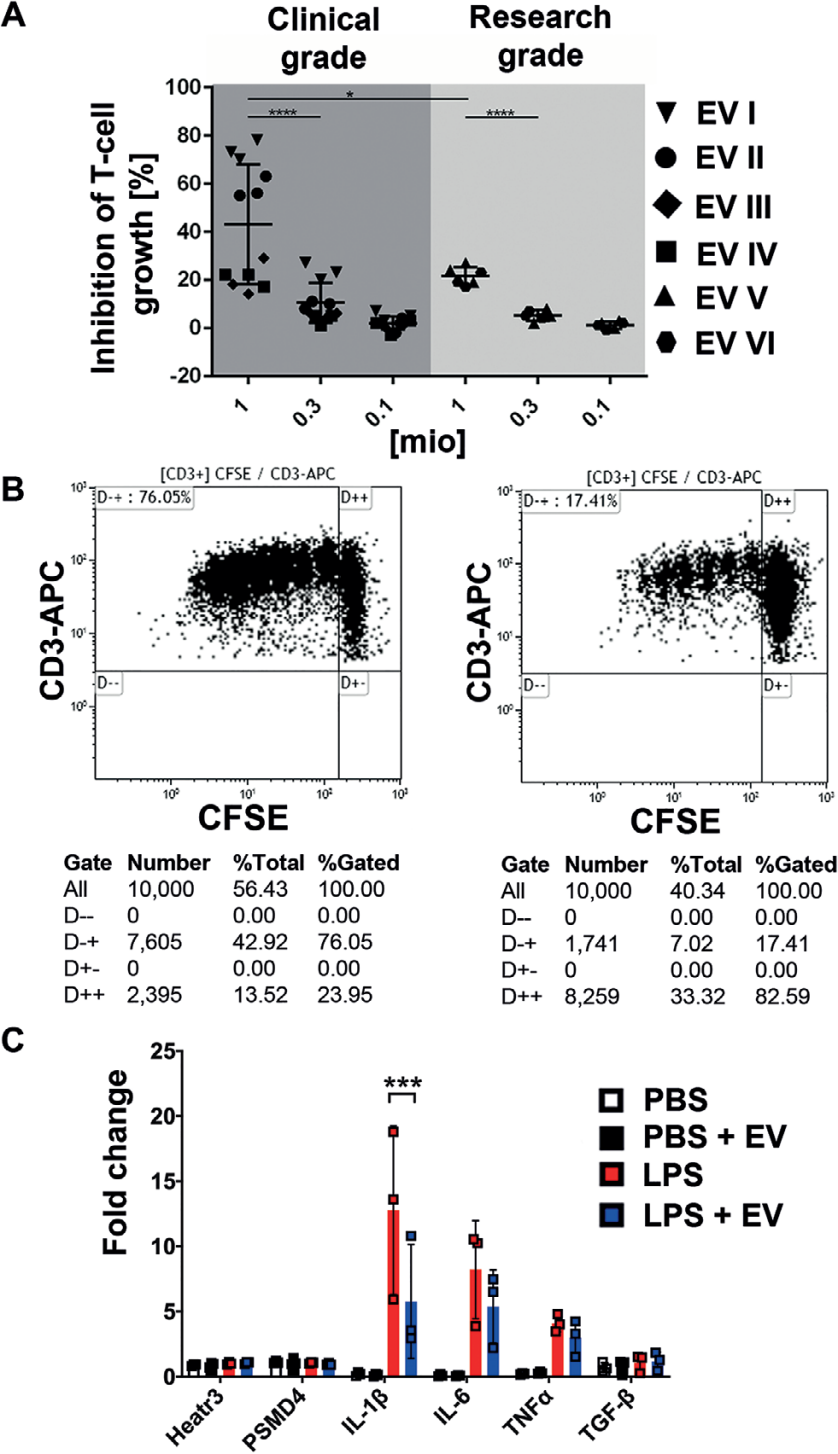
Immunomodulatory potential of mesenchymal stromal cell‐derived extracellular vesicles (MSC‐EVs). A, Four clinical‐grade (I‐IV) and two research‐grade (V and VI) batches of MSC‐EVs were tested for their capacity to inhibit phytohemagglutinin (PHA)‐induced T‐cell proliferation in dilution series as indicated (1, 0.3, 0.1 million particles per 5 × 10^5^ mononuclear cells). To determine the percentage of inhibition of carboxyfluorescein succinimidyl ester (CFSE)‐labeled CD3 T‐cell proliferation, samples were analyzed by flow cytometry in triplicates, and one‐way ANOVA was used for statistical analysis (*P < .05; ****P < .0001). Results are shown as mean ± standard deviation. B, Representative dot plots show CD3^+^ T‐cell proliferation kinetics without inhibition in the absence (left), or with inhibition in the presence of MSC‐EVs (right), respectively. For normalization, the standard stimulation (PHA only, left dot plot, upper left quadrant) was assigned to a value of 100% and the percentage of inhibition was calculated with 10 000 CD3^+^ T cells gated per analysis. C, The treatment of BV‐2 microglial cells with lipopolysaccharides (LPS) rapidly upregulates the gene expression of the proinflammatory cytokines IL‐1β, IL‐6, and TNFα. The presence of MSC‐EVs significantly reduced the induction of IL‐1β expression in BV‐2 cells in response to LPS (fold change is normalized to the endogenous controls Heatr3 and PSMD4)

In a T‐cell proliferation assay, various clinical‐grade and research‐grade EV batches exhibited immunomodulatory potential in vitro (Figure 3A). Phytohemagglutinin‐stimulated T‐cell proliferation was inhibited by MSC‐derived EVs in a dose‐dependent manner. The FACS dot plots of one representative sample showed reduced CD3^+^ T‐cell proliferation in the presence of MSC‐EVs (Figure 3B). The capacity of MSC‐EVs to interact with microglia and to modulate their phenotype was addressed in vitro using a microglial cell line (BV‐2).^50^ Activation of BV‐2 cells with LPS rapidly and strongly upregulates the expression of the proinflammatory cytokine genes IL‐1β, IL‐6, and TNFα (Figure 3C). The presence of EVs derived from UC‐MSC significantly reduced the induction of IL‐1β gene expression in BV‐2 cells in response to LPS. Importantly, the application of MSC‐EVs on cultured microglia did not elicit an activation response. Thus, EVs may directly interact with microglia during their early activation response and modify the profile of cytokine expression toward a milder inflammatory status. This was further substantiated by the analysis of activation of NF‐κβ signaling pathway in BV‐2 cell cultures, based on the phosphorylation level of NF‐κβ p65, upon LPS stimulation in the presence or absence of MSC‐EVs (Figure S5). The accumulation of phosphorylated p65 was readily visible 2 hours after LPS induction (Figure S5B), whereas in the presence of MSC‐EVs, the level of p65 phosphorylation was significantly diminished by approximately 18% (Figure S5D), as compared to the BV‐2 cells exposed to LPS only (Kruskal‐Wallis, followed by Dunn's multiple comparison, adjusted *P* < .001; Figure S5F).

### 3.3 Increased survival of SGNs is mediated by clinical‐grade EV preparations in vitro

**FIGURE 4:**
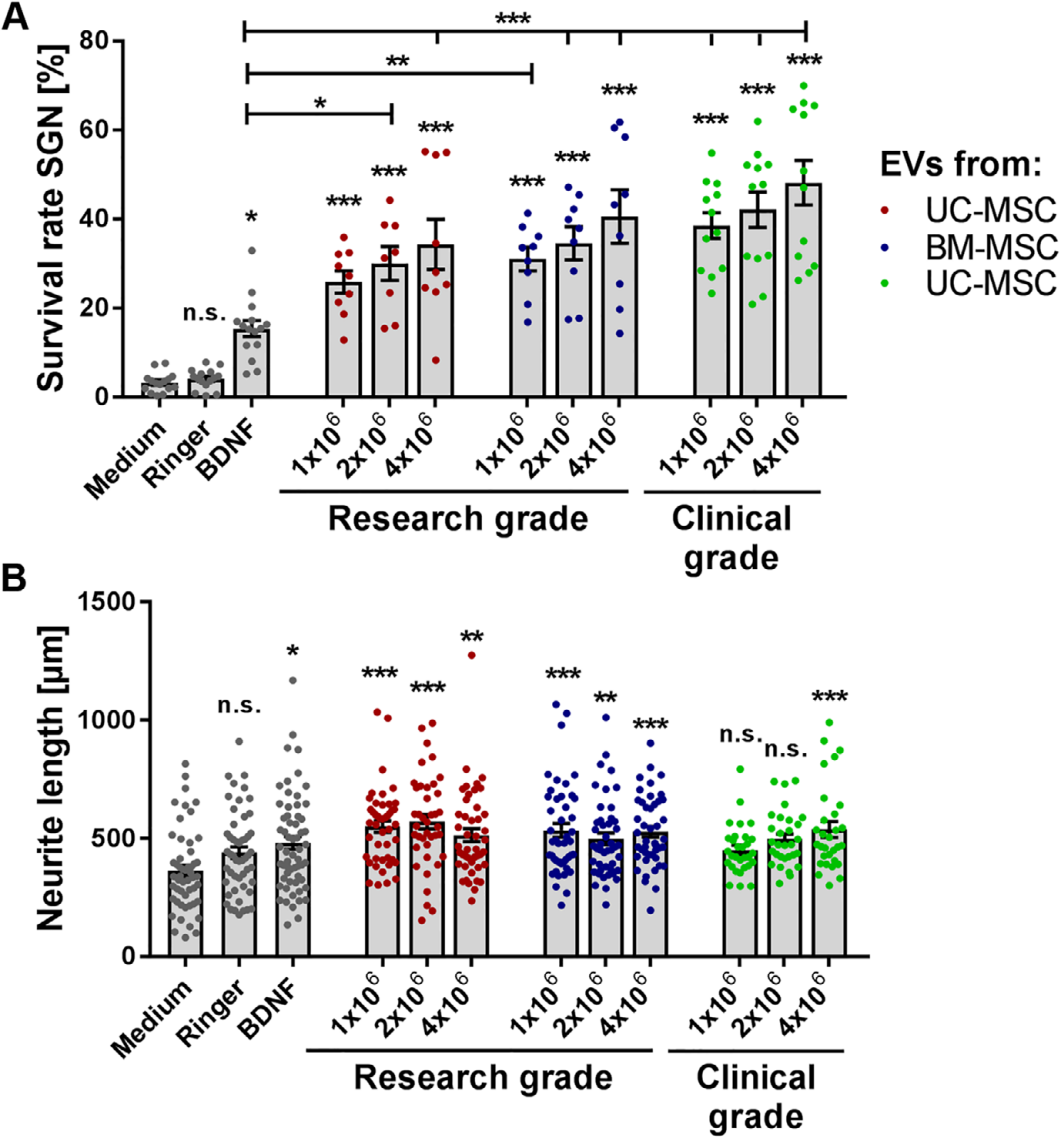
Mesenchymal stromal cell‐derived extracellular vesicles (MSC‐EVs) mediate increased survival of spiral ganglion neurons (SGN). A, Escalating doses of research‐grade EVs derived from umbilical cord and bone marrow‐derived mesenchymal stromal cells (UC‐ and BM‐MSC) as well as clinical‐grade EVs derived from UC‐MSC significantly increase the survival rate of SGN in a dose‐responsive but source‐independent manner. The highest neuroprotective activity of MSC‐EVs in this particular experiment is observed for clinical‐grade UC‐MSC‐EV preparations when compared to brain‐derived neurotrophic factor (BDNF). B, Neurite length from surviving SGN was increased in the presence of MSC‐EVs when compared to the medium control, but not in comparison to BDNF. Number of experiments: N = 3, number of replicates per experiment: n = 3 (research‐grade EVs); N = 2, n = 6 (clinical‐grade EVs); data are shown as mean ± standard error of the mean; levels of significance are shown as ***P < .001; **P < .01; *P < .05; significance levels indicated above individual bars show comparison with medium (negative control), significance levels in comparison to the positive control BDNF are separately depicted by horizontal lines. Each data point represents the determined survival rate (A) of a single well or the measured neurite length (B) of one neuron

We analyzed the effect of escalating doses of BM‐ and UC‐MSC‐EVs from 1 × 10^6^, 2 × 10^6^, and 4 × 10^6^ cells on the survival rate of SGN. BDNF was used as a positive control because of its established neuroprotective effects on SGN.^47^ When compared to 50 ng/mL BDNF, the application of all research‐grade EV preparations significantly increased the survival rate of SGN in a dose‐dependent manner (Figure 4A). The protective effect on SGN was independent of the tissue of origin of MSCs and even increased in the presence of clinical‐grade EV preparations. Medium control describes the minimal medium of the SGN without any additional treatment and in which the SGN are resuspended and seeded. Ringer is the solvent control of the EVs, as these were solved in Ringer's lactate. Treatment with mock EVs (produced from nonconditioned MSC medium submitted to TFF and ultracentrifugation) did not significantly increase the survival rate and the neurite length of SGN (Figure S6). We further measured the neurite length of the five SGNs with the longest neurites per well. The negative control (Ringer's lactate) did not alter the neurite length, whereas 50 ng/mL BDNF significantly increased the neurite length. The presence of low doses of research‐grade EVs (1‐2 × 10^6^ cells) markedly increased neurite length when compared to the medium control independent from the cell source. However, there was no clear dose‐response correlation of neurite outgrowth observed either with research or with clinical‐grade EV preparations (Figure 4B).

### 3.4 Human MSC‐derived EV preparations alter the morphology of rat SGNs and promote neurite outgrowth

**FIGURE 5:**
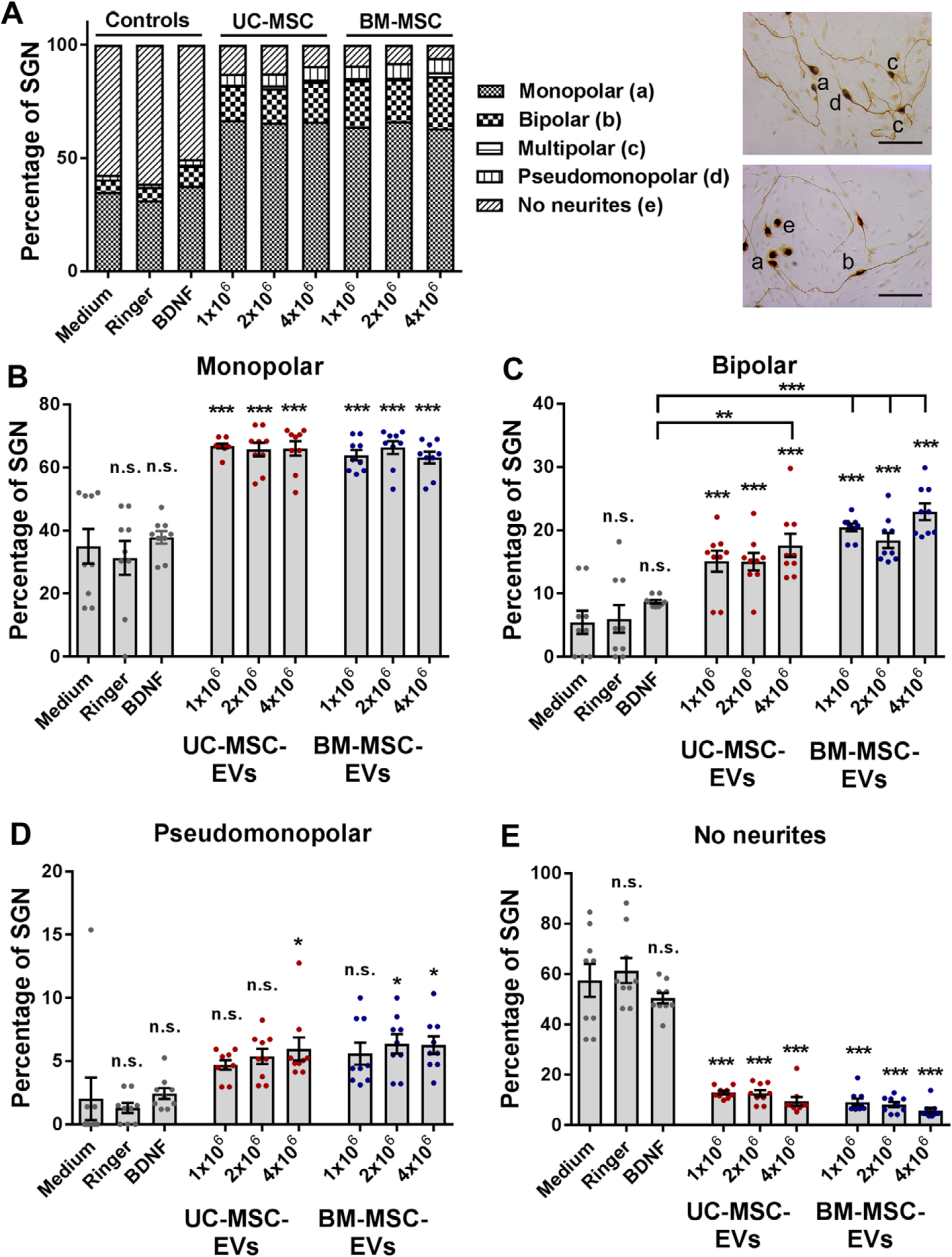
Mesenchymal stromal cell‐derived extracellular vesicles (MSC‐EVs) alter the morphology of spiral ganglion neurons (SGN). A, The various morphologies of SGN treated with research‐grade EV preparations derived from umbilical cord and bone marrow‐derived mesenchymal stromal cells (UC‐ and BM‐MSC) are depicted in an overview graph (left). Representative images of SGN with mono‐, bi‐, multi‐, pseudomonopolar morphology, and SGN lacking neurites are shown (right) and designated as (a) to (e). B, The percentage of monopolar neurons increases in SGN treated with all EV preparations and concentrations. C, The highest concentration of the UC‐MSC‐EVs and all tested BM‐MSC‐EV doses clearly increase the percentage of bipolar neurons in comparison to brain‐derived neurotrophic factor (BDNF). D, The percentage of pseudomonopolar neurons increases after the treatment with high concentrations of EVs. E, The number of SGN lacking neurites is significantly reduced in the presence of MSC‐EVs. Scale bar: 100 μm; number of experiments: N = 3, number of replicates per experiment: n = 3. ***P < .001; **P < .01; *P < .05; significance levels indicated above individual bars show comparison with medium (negative control), significance levels in comparison to the positive control BDNF are separately depicted by horizontal lines. Each data point represents the determined percentage of SGN in a single well (B‐E)

Neuronal morphologies of SGN can be categorized as monopolar, bipolar, multipolar, pseudomonopolar, and neurons without neurites. The number of counted neurons of each category was related to the total number of SGN per well. The relative occurrence of the morphological subclasses that became discernible after the different treatments is shown in an overview graph (Figure 5A, left) and representative images of the different neuronal categories are depicted (Figure 5A, right). Analysis of the morphology and assignment to one of the morphological subclasses of the SGN revealed significant differences between the control treatments and treatment with escalating doses of either BM‐ or UC‐MSC‐EVs. The monopolar neurons represent the most prevalent type among all counted SGNs. In comparison to the negative controls (medium: 35.03 ± 5.47% and Ringer's lactate: 31.31 ± 5.36%) and the positive control (BDNF: 37.83 ± 2.01%), there was a significant and dose‐independent increase of the monopolar neuronal fraction after treatment with MSC‐EVs (mean value 66.21 ± 1.77%, *P* ˂ .001; Figure 5B). The highest concentration of the UC‐MSC‐EVs and all tested BM‐MSC‐EV doses clearly increased the percentage of bipolar neurons in comparison to BDNF (Figure 5C). The percentage of pseudomonopolar neurons is generally below 10%. However, the percentage significantly increased after treatment with high concentrations of MSC‐EVs when compared to the control conditions (Figure 5D). Multipolar neurons represent only a small fraction of the neuronal population (below 2%), and we observed no differences when compared to the controls (data not shown). By contrast, the number of SGNs with no neurites was significantly decreased by treatment with MSC‐EVs (mean value 11.64 ± 1.25%) when compared to control conditions (BDNF: 50.47 ± 2.10%, medium: 57.50 ± 6.51%, Ringer's lactate: 61.41 ± 4.93%, *P* ˂ .001; Figure 5E).

### 3.5 Noise trauma can be alleviated by treatment with MSC‐EVs in a murine in vivo model

**FIGURE 6:**
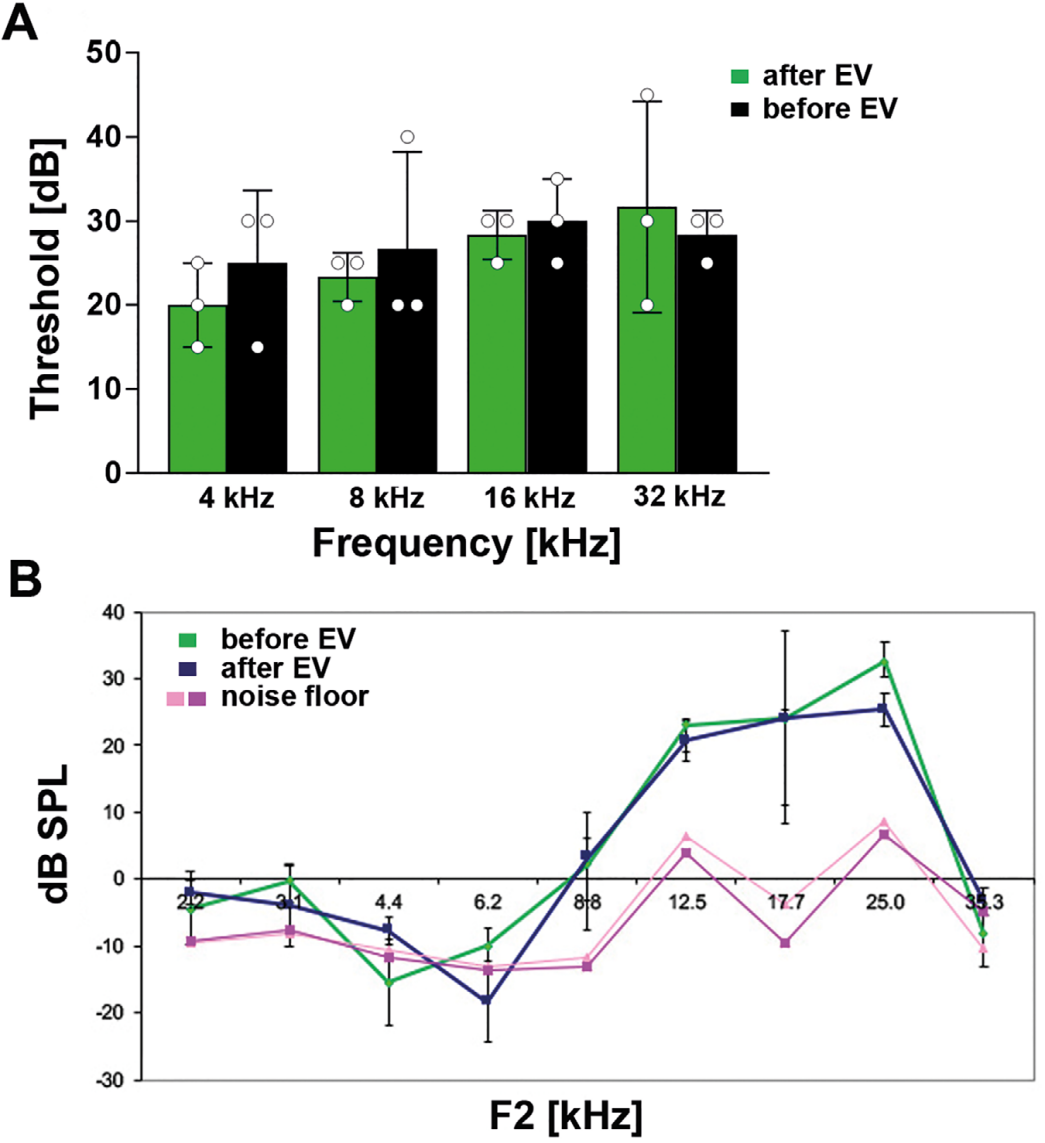
Extracellular vesicles’ (EVs) delivery does not impair physiological hearing in vivo. A, The intracochlear delivery of 1 μL of EVs from umbilical cord‐derived mesenchymal stromal cells (UC‐MSCs) to normal hearing mice does not negatively affect hearing as shown by auditory brainstem response (ABR) thresholds (green) when compared to the ABR before the EV treatment (black). B, The outer hair cell function evaluated by distortion product otoacoustic emission (DPOAE) testing is also not impaired in response to EV delivery (green line) when tested at day 5 postdelivery (blue line) and compared to the DPOAEs before EV application. Pink and rose lines represent the noise floor. Abbreviations: dB, decibel; F, frequency; kHz, kiloHertz; SPL, sound pressure level

**FIGURE 7:**
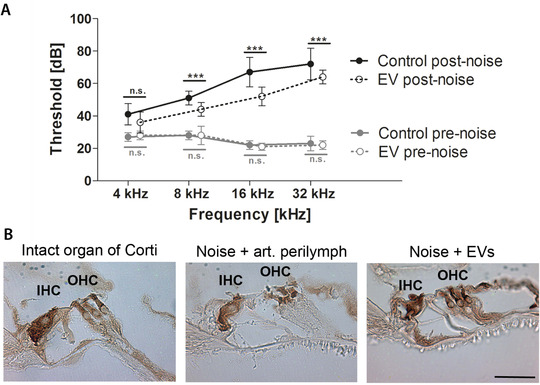
Protective effects of mesenchymal stromal cell‐derived extracellular vesicles (MSC‐EVs) in a mouse noise trauma model in vivo. A, Mean auditory brainstem response (ABR) thresholds (in dB) are plotted for the different frequencies (4, 8, 16, 32 kHz) and designated as pre‐noise (grey) or post‐noise values (black, 4 weeks after noise trauma). Treatment with MSC‐EVs on day 3 post‐noise (n = 5) attenuates threshold shifts after noise trauma when compared to the control (artificial perilymph, n = 5) treated animals, especially in the higher frequencies. Pre‐noise ABR thresholds of all animals tested are comparable and at physiological levels. Data are presented as mean ± standard deviation; ***P ˂ .001, n.s., not significant. B, Representative images after immunohistochemical staining for myosin VIIa are shown. A normal organ of Corti (no noise trauma, no treatment, displays intact inner and outer hair cells (IHC and OHC), while noise trauma and treatment with control (artificial perilymph) results in intact inner but damaged outer hair cells. Post‐noise treatment with EVs from umbilical cord (UC)‐derived MSCs rescues the organ of Corti with intact inner and outer hair cells. Scale bar: 50 μm applies to all images shown

To determine if the MSC‐derived EV preparations induced any toxicity in the ear, normal hearing mice were treated with 1 μL of EVs (containing 2 × 10^10^ particles/mL) from UC‐MSCs. The mice showed no signs of vestibular dysfunction or head tilt postoperatively. Evaluation of hearing 5 days after the delivery of EVs demonstrated that there were no changes in ABR thresholds (Figure 6A). Additionally, outer hair cell function determined by DPOAE was unaffected (Figure 6B). Next, we evaluated the effect of the MSC‐EVs in vivo as treatment for noise‐induced hearing loss in mice. The ABR thresholds of all animals were on a comparable normal level at the beginning (EVs and control pre‐noise; Figure 7A). The noise trauma was induced with a sinusoidal tone at 16 kHz: 118 dB for 3 hours. This level of noise causes significant damage especially to the apical high‐frequency region of the cochlea. At day 3 post‐noise trauma, MSC‐EVs or artificial perilymph (control group) was administered. The treatment with EVs attenuated threshold shifts when compared to the control treated mice, especially in the higher frequencies (EVs and control post‐noise; Figure 7A). At 4 weeks post‐noise trauma, histological sections showed degeneration of outer hair cells in the control group (artificial perilymph in contrast to organs of Corti from the EV‐treated group, Figure 7B). The EV treatment resulted in intact outer hair cells post‐noise trauma similar to those of healthy control animals (without any treatment, Figure 7B).

## 4 DISCUSSION

We provide first in vivo evidence that human UC‐MSC‐EV preparations have the potential to rescue hearing after noise trauma, are nontoxic, prevent noise‐damaged mouse hair cells from degeneration, and protect primary rat auditory neurons in vitro. When compared to the current best‐in‐class neuroprotective factor BDNF, the treatment with UC‐MSC‐EVs significantly increased the survival rate of SGN in vitro. This may be due to the immunomodulatory effects of MSC mediated by their EVs as has been also shown for poststroke neuronal regeneration of the brain.^23^, ^32^ Furthermore, BDNF appears to be one of the key factors in mediating this effect. We have shown here that MSC‐EVs have the capacity to potentiate protective and neuroregenerative effects by yet to be defined mechanisms. Proteins, lipids, nucleic acid components, and various other factors may influence the biologic effects of MSC‐EV preparations.

Immunomodulatory cytokines and chemokines have been identified in the supernatant of mononuclear cells derived from bone marrow, indicating that these factors may mediate the survival of auditory neurons.^16^ In addition, the cytokine erythropoietin was shown to modulate the effects of BDNF and the TGF‐ß superfamily member activin A, thereby improving the neuroprotective effect on auditory neurons in vitro.^10^ Comparative analysis of cytokines and chemokines in MSC‐EV preparations showed that very low amounts of most proinflammatory cytokines combined with moderate levels of IL‐6, IL‐8, and BDNF could be involved in the protective effect exhibited by MSC‐EVs. Indeed, interactions between BDNF and cytokines have been demonstrated for the central nervous system^51^ and could also be mediated by EVs. Combined effects of EVs and cytokines may differ from their independent effects.^52^ Thus, EVs could not only modify the effects of inflammatory cytokines,^52^ but also of other soluble mediators, leading to therapeutic effects that could not be achieved by the individual factors alone. However, such interactions have not been investigated in the inner ear hitherto. In a previous study, we showed that biological therapies based on platelet‐rich plasma mediate their effects via pathways regulating inflammation and immune responses such as the p38 mitogen‐activated protein kinase and the NF‐κB pathway.^13^ Regulated by NF‐κB is the TNFα‐induced secretion of IL‐8.^53^, ^54^ The transcription factor sterol regulatory element‐1 (SREBF1) that interacts with CREB1 thereby regulating the gene expression of IL‐8^52^ may be responsible for the EV‐induced effects. Indeed, we found an upregulation of SREBF1 in the cochlea after treatment with UC‐MSCs possibly mediating the protective effects in the noise‐injured inner ear (unpublished results).

Intracellular signaling is also affected by tetraspanins. Multiplex surface profiling of MSC‐EVs revealed the exposure of the canonical α5ß1 fibronectin receptor (CD29/CD49e) as well as the hyaluronic acid receptor CD44. Tetraspanins CD9, CD63, and CD81 have been shown to activate integrin ß1 and other integrins, suggesting a cooperative activity of these molecules in the membrane of EVs. Our data support the notion that tetraspanin‐rich microdomains that are naturally present in EVs are distributed into the plasma membrane of the target cells upon EV fusion and influence their ability to interact with the extracellular matrix (ECM) and to interpret ECM‐derived signals. Fibronectin is considered an important substrate for regenerative outgrowth of peripheral nerves^55^ and plays a role in the modulation of inner ear spiral ganglion neurite outgrowth.^56^ Based on their specific composition, human stromal cell‐derived EVs may modify cellular signal transduction, more specifically the mechanotransduction of the target cells, resulting in the restoration or rescue of hair cell function. In our experimental setting, even 3 days after induction of hair cell damage by noise trauma, hearing restoration by the rescue of hair cells was possible due to treatment with the biologically active EV preparations. Moreover, tetraspanins are known to regulate cell morphology.^57^

Indeed, application of MSC‐EVs affected the morphology of the surviving neurons in the in vitro experiments: the number of monopolar and especially bipolar neurons was increased and the number of SGN with no neurites was significantly decreased. The in vivo morphology of type I SGN, which connect the inner hair cells with the brainstem and that contribute to the transmission of the auditory signal from external to the central brain region, is bipolar.^58^, ^59^ Our investigations demonstrated that MSC‐EVs particularly increased the number of bipolar neurons, representing the physiologically relevant morphology of SGN. By contrast, the number of SGN with no neurites was reduced by the treatment with EVs, indicating a potential to increase neuritogenesis. The influence of specific factors on the morphology of cultured auditory neurons has been investigated in other studies demonstrating that the administration of a single factor such as BDNF, leukemia inhibitory factor (LIF), or LIF‐type cytokines increased the number of SGN in terms of the percentage of bipolar neurons.^49^, ^60^ A later study revealed that the combined treatment of BDNF and the LIF‐type cytokine ciliary neurotrophic factor increased the percentage of bipolar neurons in a synergistic manner.^12^ Our results confirm these observations, since only the treatment with the MSC‐EVs but not with recombinant BDNF alone significantly increased the number of survived bipolar neurons. The concentration of the recombinant BDNF was in our in vitro investigations 50 ng/mL. However, the measured BDNF in one of our EV preparations was only approximately 2 ng/mL. In a previous study, we have already shown that cell‐derived BDNF (from genetically modified cells) is more potent than recombinant BDNF, and thus lower BDNF amounts (9.09 ng/mL) are sufficient for increased survival of SGN.^61^

The observed synergistic effect between EVs, and also of EVs and BDNF (and presumably also other factors) in the potentiation of BDNF‐induced neuronal survival suggests the involvement of multiple factors for the cyto‐protective effects in the inner ear. A potential mechanism for such a synergy may reside in the intrinsic intracellular signaling cascades of BDNF and its receptors TrkB and TrkA2a. The latter receptor is activated by adenosine that in turn transactivates TrkB and enhances the promotion of neuronal survival.^62^ CD73 is a 5`‐ectonucleotidase that specifically cleaves adenosine monophosphate to release adenosine. CD73 is highly abundant in the membrane of MSC‐derived EVs and surface profiling has confirmed the presence of CD73 in the MSC‐EV preparations. The activity of CD73 on EV surface and the subsequent release of adenosine could potentiate the effect of the low levels of BDNF present in the EV preparations and result in the observed neuroprotection. Adenosine is an extracellular messenger and has been shown to protect the cochlea from oxidative stress as a result from noise trauma.^63^, ^64^ CD73 is expressed in the rat cochlea in the stria vascularis, the spiral ligament and the SGN alongside with members of the ecto‐nucleoside triphosphate diphosphohydrolase family.^65^ They might be involved in the regulation of cochlear sensitivity by hydrolysing ATP to adenosine thereby protecting from excessive activation of ATP‐gated channels, which may trigger cytotoxicity.^65^ Whether this mechanism is involved in the protection of hearing and hair cells by EVs after noise trauma requires further investigation. A recently published study showed that exosome‐associated heat shock 70‐kDa protein (HSP70) seems to be one of the key factors in mediating hair cell survival in the presence of ototoxic drugs such as neomycin.^30^ However, the underlying mechanism is not fully understood but seems to be induced through the activation of Toll‐like‐receptor‐4 (TLR4) on the hair cells. In cardiomyocytes, the protective effect of exosomal HSP70 against ischemia/reperfusion injury was mediated via the activation of ERK1/2 and p38 MAPK,^66^, ^67^ two pathways that can also be activated by BDNF.

Analyses of the miRNA content of clinical‐ and research‐grade EV preparations identified several miRNAs that may support the observed effects. Homo sapiens (hsa) miR146a‐5p participates in regulatory T‐cell (T‐regs)‐directed suppression. In EVs derived from T‐regs, a high miR146a‐5p content has been identified that can aid in the suppression of pathological Th1 activation.^68^ In addition, miR146a reduces the proinflammatory signaling in human adipocytes^69^ and is upregulated in the EVs derived from the choroid plexus upon induction of robust inflammatory responses via lipopolysaccharids.^70^ MiR21‐5p, also found among the top‐upregulated miRNAs of MSC‐EVs, is expressed at higher levels in T‐regs, which control autoimmune response and inhibit autoimmunity.^68^ Another prominent miRNA found in UC‐MSC‐EV preparations is hsa miR148a‐3p, a repressor of the NF‐κB signaling and inflammatory gene expression.^71^ RNA‐based signature profiles have been used by others to discriminate therapeutically potent from nonpotent EVs for the protection from myocardial infarction.^72^ Whether the miRNAs identified in our EV preparations can be used as surrogate markers to predict a therapeutic potency of UC‐MSC‐EVs has to await further detailed functional studies.

One limitation associated with the herein presented study is the impact of the isolation method on the efficacy of EVs. The EVs in the present study were isolated by serial ultracentrifugation steps. With this isolation procedure, overabundant soluble plasma proteins may not be discarded.^73^ Indeed, coisolation of extracellular protein‐RNA complexes that overlap in size with EVs has been not only discussed to be possibly involved in mediating the effects of EVs^74^ but also in altering the effects of EVs. For example, immune‐relevant stimuli may not only induce changes in the RNA content^74^ of EVs but may also modulate their efficacy profile.^52^ Whether the effects observed in vitro and in vivo in the present study are due to the applied EVs or due to the combined effects of EVs and cytokine or miRNA profiles need further investigation. It would also be interesting to assess the in vivo localization of EVs after administration into the inner ear and their persistence despite challenges in the labeling of EVs.^40^, ^75^, ^76^

The present study provides initial proof of the high potential of UC‐MSC‐derived EVs to support neuronal survival and to repair noise‐induced damage in the inner ear. The capacity to manufacture and characterize clinical‐grade EVs under current GMP will support the rapid translation of these research findings into clinical application and may be helpful also for the intervention in other organ systems and disease indications. Even though more research into the mechanism of action of MSC‐EVs is needed, our data indicate that clinical grade EVs derived from UC‐MSC are highly protective for auditory neurons and can protect the inner ear against noise trauma in vivo. These findings provide a solid foundation for the future use of human stromal cell‐derived EVs as a novel cell‐free therapeutic approach for the protection of the inner ear.

## CONFLICT OF INTEREST

Eva Rohde is the CEO of PMU Innovations GmbH (Salzburg) and Medical Consultant of MDimune Inc. (Seoul, Korea). Mario Gimona is the Consulting Chief Manufacturing Officer of MDimune Inc. (Seoul, Korea).

## AUTHOR CONTRIBUTIONS

Athanasia Warnecke and Jennifer Harre: Conception/design, financial support, performing the in vitro experiments, collection and/or assembly of data, data analysis and interpretation, manuscript writing, and final approval of the manuscript. Hinrich Staecker: Performing the in vivo experiments, collection and/or assembly of data, data analysis and interpretation, and final approval of the manuscript. Nils Prenzler: Collection and/or assembly of data, final approval of the manuscript. Dirk Strunk, Sebastien Couillard‐Despres, and Pasquale Romanelli: In vitro testing and final approval of the manuscript. Julia Hollerweger and Teresa Lassacher: EV manufacturing under good manufacturing practice (GMP) and in vitro testing. Daniela Auer: In vitro testing, quality control under GMP. Karin Pachler: EV manufacturing and in vitro testing. Georg Wietzorrek: Data analysis and final interpretation, and final approval of the manuscript. Ulrike Köhl: Handling and storage of clinical EV preparations under GMP and good distribution practice, and final approval of the manuscript. Thomas Lenarz: Administrative support, manuscript writing, and final approval of the manuscript. Katharina Schallmoser: pHLP manufacturing GMP and final approval of the manuscript. Christine S. Falk: Performance of the Luminex‐based multiplex protein arrays, data analysis, final approval of the manuscript. Sandra Laner‐Plamberger: Primary stromal cell isolation, in vitro testing, and final approval of the manuscript. Eva Rohde: Conception and design, administrative support, manuscript writing, and final approval of the manuscript. Mario Gimona: Conception and design of EV manufacturing strategy, EV manufacturing, financial and administrative support, manuscript writing, and final approval of the manuscript.

## Supporting information

Supporting Information
