# Supplementary material for: Extracellular vesicles from human multipotent stromal cells protect against hearing loss after noise trauma in vivo
Source: Clin Transl Med. 2020 Dec 21;10(8):e262. doi: 10.1002/ctm2.262 (PMC7752163; doi:10.1002/ctm2.262)
Supplement: Supplementary file 1 — Supporting Information [file CTM2-10-e262-s001.docx]

**SUPPLEMENTAL MATERIALS AND METHODS**

***Immunophenotyping and determination of parental cell viability by flow cytometry***

Immunophenotype and viability analysis of MSC was carried out according to the suggested marker profile for defining MSC identity as published by the International Society of Cell Therapy (ISCT) in 2005. In brief, collected cells were centrifuged (300 × g for 6 min), resuspended in 5 % v/v sheep serum-containing blocking buffer to reach a concentration of 1.5 x 10^7^cells/mL and incubated for 20 min at +4 °C in the dark. 3 x 10^5^ cells were stained with mouse anti-human monoclonal antibodies against CD90 (IM1839U, Beckman Coulter, France), CD105 (MCHD10505, Life Technologies, Austria), CD14, CD34, CD45, CD73, HLA-II (DR) (345785, 345802, 345808, 550257, 347400 Becton Dickinson, Austria), or with corresponding isotype controls (345815, 345818, 555743, 345816, 349051, Becton Dickinson, Austria) for 25 min at +4 °C in the dark. Thereafter, samples were washed with cold PBS and resuspended in 100 µL 7AAD-containing PBS (1:50 dilution, 0.0005 w/v % final concentration) and stained for 10 min at room temperature protected from light. Finally, 400 µL cold PBS was added and the samples were measured immediately using FACSCanto II flow cytometer (Becton Dickinson) until 10.000 events were recorded per staining. Blue (488 nm) and red (633 nm) laser excited fluorescence signals were detected with the following standard light filters: FITC: 530/30 nm; PE: 585/42 nm; APC: 660/20 nm; 7AAD: 670LP. Results were analysed with FlowJo 10.2 (FlowJo LLC, Ashland, Oregon, USA). FSCA -SSCA dot plot analyses were applied for debris exclusion, and a doublet discrimination panel was set on the FSC channel for the detection of height and width of the fluorescence signals. The ratio of the viable cells was determined on [SSC-7AAD] dot plot.

**MACSPlex surface protein profiling**

The MACSPlex Exosome Kit (Miltenyi Biotec) is a bead-based multiplexed FACS-based assay for the analysis of surface markers present on EVs. We have used the MACSPlex kit according to the manufacturer´s instruction and following a validated standard operating procedure with 5 x 10^7^ to 5 x 10^8^ particles as input. Data acquisition was conducted on a FACS Canto II (BD Biosciences). For additional CD73 analysis an anti-CD73-BV421 antibody (BD Biosciences) was added to the CD9/CD63/CD81 cocktail. Isotype control normalization was performed and data normalization was directed towards CD9/CD63/CD81 APC signal.

**In vitro tri-lineage differentiation assays.**

For osteogenic and adipogenic in vitro differentiation, 1 x 10^3^ UC-MSCs per cm2 (passage 2) were seeded in 12-well plates. After 24 hours, the medium was replaced by either osteogenic or adipogenic differentiation medium as described (Laner-Plamberger et al., 2015). UC-MSCs cultured in normal HPL-based growth medium served as control. At day 21, cells were stained after fixation with 4% paraformaldehyd (PFA, Sigma Aldrich) either with 0.5% Alizarin Red (Sigma Aldrich) or 1% Sudan III (Sigma Aldrich). Chondrogenic in vitro differentiation was induced using 5x10^5^ pelleted UC-MSCs (passage 3) cultivated in hMSC chondrogenic SingleQuots (Lonza, Basel, Switzerland) in the presence of TGF-β3 (20 µg/ml) for 21 days. Cell pellets were processed as described (Laner-Plamberger et al., 2015) and stained in 1% Alcian Blue staining solution (8GS, Gatt-Koller, Absam, Austria) and Nuclear Fast Red solution (Sigma Aldrich). Photographs were done using a PrimoVert Light microscope and an AxioCam ERc5s digital camera (both Zeiss, Germany).

**SUPPLEMENTAL FIGURES AND TABLES**

| **Table S1**  **Flow cytometric surface marker profile of a representative umbilical cord-MSC batch at the time of EV harvest.** | | | |
| --- | --- | --- | --- |
| **Marker** | **Result (%)** | **Criteria** |  |
| CD45 | 0.17 | ≤ 2 % |  |
| CD19 | 0.14 | ≤ 2 % |  |
| HLA-DR | 0.03 | ≤ 2 % |  |
| CD34 | 0.15 | ≤ 2 % |  |
| CD49e | 99.3 | ≥ 95% |  |
| CD14 | 0.02 | ≤ 2 % |  |
| CD29 | 99.8 | ≥ 95% |  |
| CD105 | 99.8 | ≥ 95 % |  |
| CD44 | 99.5 | ≥ 95% |  |
| CD90 | 98.3 | ≥ 95 % |  |
| CD166 | 99.5 | ≥ 95% |  |
| CD73 | 99.4 | ≥ 95 % |  |
|  |  |  |  |
| Viability at time of harvest | 95.5 | ≥ 95% |  |
|  |  |  |  |

| **Table S2**  **Micro-RNA Profiling identifies three miRNAs among the top results in individual EV samples**  The top 20 miRNA results are shown. The three miRNAs highlighted in green were found among the top 7 position in the respective samples derived from three individual donors of UC-MSC-EVs.   \|  \|  \|  \|  \|  \|  \|  \| \| --- \| --- \| --- \| --- \| --- \| --- \| --- \| \|  \| **UC-MSC-EV LA0013-1** \|  \| **UC-MSC-EV HJ16B** \|  \| **UC-MSC-EV HS15B** \|  \| \| **Rank** \| hsa-miRNA \| counts \| hsa-miRNA \| counts \| hsa-miRNA \| counts \| \| **1** \| **hsa-miR-146a-5p** \| 3453739 \| [**hsa-miR-146a-5p**](http://www.exiqon.com/ls/Pages/DeepLink.aspx?search_area=tpt&search_string=hsa-miR-146a-5p&search_type=mirna) \| 14639 \| [**hsa-miR-146a-5p**](http://www.exiqon.com/ls/Pages/DeepLink.aspx?search_area=tpt&search_string=hsa-miR-146a-5p&search_type=mirna) \| 6088 \| \| **2** \| hsa-miR-92a-3p \| 1873720 \| [hsa-miR-221-3p](http://www.exiqon.com/ls/Pages/DeepLink.aspx?search_area=tpt&search_string=hsa-miR-221-3p&search_type=mirna) \| 4947 \| [**hsa-miR-148a-3p**](http://www.exiqon.com/ls/Pages/DeepLink.aspx?search_area=tpt&search_string=hsa-miR-148a-3p&search_type=mirna) \| 3890 \| \| **3** \| hsa-miR-22-3p \| 1264079 \| [**hsa-miR-21-5p**](http://www.exiqon.com/ls/Pages/DeepLink.aspx?search_area=tpt&search_string=hsa-miR-21-5p&search_type=mirna) \| 4486 \| [**hsa-miR-21-5p**](http://www.exiqon.com/ls/Pages/DeepLink.aspx?search_area=tpt&search_string=hsa-miR-21-5p&search_type=mirna) \| 3632 \| \| **4** \| hsa-miR-30d-5p \| 984508 \| [hsa-miR-92a-3p](http://www.exiqon.com/ls/Pages/DeepLink.aspx?search_area=tpt&search_string=hsa-miR-92a-3p&search_type=mirna) \| 4074 \| [hsa-miR-320a](http://www.exiqon.com/ls/Pages/DeepLink.aspx?search_area=tpt&search_string=hsa-miR-320a&search_type=mirna) \| 2246 \| \| **5** \| hsa-miR-221-3p \| 642625 \| [hsa-miR-100-5p](http://www.exiqon.com/ls/Pages/DeepLink.aspx?search_area=tpt&search_string=hsa-miR-100-5p&search_type=mirna) \| 3976 \| [hsa-miR-92a-3p](http://www.exiqon.com/ls/Pages/DeepLink.aspx?search_area=tpt&search_string=hsa-miR-92a-3p&search_type=mirna) \| 2160 \| \| **6** \| **hsa-miR-148a-3p** \| 543104 \| [**hsa-miR-148a-3p**](http://www.exiqon.com/ls/Pages/DeepLink.aspx?search_area=tpt&search_string=hsa-miR-148a-3p&search_type=mirna) \| 3390 \| [hsa-let-7f-5p](http://www.exiqon.com/ls/Pages/DeepLink.aspx?search_area=tpt&search_string=hsa-let-7f-5p&search_type=mirna) \| 2058 \| \| **7** \| **hsa-miR-21-5p** \| 542563 \| [hsa-miR-320a](http://www.exiqon.com/ls/Pages/DeepLink.aspx?search_area=tpt&search_string=hsa-miR-320a&search_type=mirna) \| 3302 \| [hsa-miR-151a-3p](http://www.exiqon.com/ls/Pages/DeepLink.aspx?search_area=tpt&search_string=hsa-miR-151a-3p&search_type=mirna) \| 1936 \| \| **8** \| hsa-miR-320a \| 431189 \| [hsa-miR-151a-3p](http://www.exiqon.com/ls/Pages/DeepLink.aspx?search_area=tpt&search_string=hsa-miR-151a-3p&search_type=mirna) \| 2964 \| [hsa-miR-100-5p](http://www.exiqon.com/ls/Pages/DeepLink.aspx?search_area=tpt&search_string=hsa-miR-100-5p&search_type=mirna) \| 1831 \| \| **9** \| hsa-miR-151a-3p \| 420053 \| [hsa-miR-584-5p](http://www.exiqon.com/ls/Pages/DeepLink.aspx?search_area=tpt&search_string=hsa-miR-584-5p&search_type=mirna) \| 2871 \| [hsa-let-7i-5p](http://www.exiqon.com/ls/Pages/DeepLink.aspx?search_area=tpt&search_string=hsa-let-7i-5p&search_type=mirna) \| 1815 \| \| **10** \| hsa-miR-24-3p \| 368814 \| [hsa-let-7f-5p](http://www.exiqon.com/ls/Pages/DeepLink.aspx?search_area=tpt&search_string=hsa-let-7f-5p&search_type=mirna) \| 2864 \| [hsa-miR-10a-5p](http://www.exiqon.com/ls/Pages/DeepLink.aspx?search_area=tpt&search_string=hsa-miR-10a-5p&search_type=mirna) \| 1698 \| \| **11** \| hsa-miR-584-5p \| 349697 \| [hsa-let-7i-5p](http://www.exiqon.com/ls/Pages/DeepLink.aspx?search_area=tpt&search_string=hsa-let-7i-5p&search_type=mirna) \| 2700 \| [hsa-miR-584-5p](http://www.exiqon.com/ls/Pages/DeepLink.aspx?search_area=tpt&search_string=hsa-miR-584-5p&search_type=mirna) \| 1632 \| \| **12** \| hsa-let-7i-5p \| 316634 \| [hsa-miR-30d-5p](http://www.exiqon.com/ls/Pages/DeepLink.aspx?search_area=tpt&search_string=hsa-miR-30d-5p&search_type=mirna) \| 2279 \| [hsa-let-7a-5p](http://www.exiqon.com/ls/Pages/DeepLink.aspx?search_area=tpt&search_string=hsa-let-7a-5p&search_type=mirna) \| 1416 \| \| **13** \| hsa-miR-30e-5p \| 285115 \| [hsa-let-7a-5p](http://www.exiqon.com/ls/Pages/DeepLink.aspx?search_area=tpt&search_string=hsa-let-7a-5p&search_type=mirna) \| 1987 \| [hsa-miR-221-3p](http://www.exiqon.com/ls/Pages/DeepLink.aspx?search_area=tpt&search_string=hsa-miR-221-3p&search_type=mirna) \| 1347 \| \| **14** \| hsa-miR-26a-5p \| 222099 \| [hsa-miR-103a-3p](http://www.exiqon.com/ls/Pages/DeepLink.aspx?search_area=tpt&search_string=hsa-miR-103a-3p&search_type=mirna) \| 1944 \| [hsa-miR-30d-5p](http://www.exiqon.com/ls/Pages/DeepLink.aspx?search_area=tpt&search_string=hsa-miR-30d-5p&search_type=mirna) \| 1051 \| \| **15** \| hsa-miR-25-3p \| 211267 \| [hsa-miR-10a-5p](http://www.exiqon.com/ls/Pages/DeepLink.aspx?search_area=tpt&search_string=hsa-miR-10a-5p&search_type=mirna) \| 1699 \| [hsa-let-7g-5p](http://www.exiqon.com/ls/Pages/DeepLink.aspx?search_area=tpt&search_string=hsa-let-7g-5p&search_type=mirna) \| 911 \| \| **16** \| hsa-miR-10a-5p \| 196328 \| [hsa-let-7g-5p](http://www.exiqon.com/ls/Pages/DeepLink.aspx?search_area=tpt&search_string=hsa-let-7g-5p&search_type=mirna) \| 1540 \| [hsa-miR-103a-3p](http://www.exiqon.com/ls/Pages/DeepLink.aspx?search_area=tpt&search_string=hsa-miR-103a-3p&search_type=mirna) \| 907 \| \| **17** \| hsa-let-7f-5p \| 194072 \| [hsa-miR-24-3p](http://www.exiqon.com/ls/Pages/DeepLink.aspx?search_area=tpt&search_string=hsa-miR-24-3p&search_type=mirna) \| 1517 \| [hsa-miR-423-5p](http://www.exiqon.com/ls/Pages/DeepLink.aspx?search_area=tpt&search_string=hsa-miR-423-5p&search_type=mirna) \| 874 \| \| **18** \| hsa-miR-199a-3p \| 173702 \| [hsa-miR-486-5p](http://www.exiqon.com/ls/Pages/DeepLink.aspx?search_area=tpt&search_string=hsa-miR-486-5p&search_type=mirna) \| 1273 \| [hsa-miR-26a-5p](http://www.exiqon.com/ls/Pages/DeepLink.aspx?search_area=tpt&search_string=hsa-miR-26a-5p&search_type=mirna) \| 763 \| \| **19** \| hsa-miR-423-5p \| 168853 \| [hsa-miR-26a-5p](http://www.exiqon.com/ls/Pages/DeepLink.aspx?search_area=tpt&search_string=hsa-miR-26a-5p&search_type=mirna) \| 1231 \| [hsa-miR-486-5p](http://www.exiqon.com/ls/Pages/DeepLink.aspx?search_area=tpt&search_string=hsa-miR-486-5p&search_type=mirna) \| 706 \| \| **20** \| hsa-miR-27b-3p \| 166726 \| [hsa-miR-134-5p](http://www.exiqon.com/ls/Pages/DeepLink.aspx?search_area=tpt&search_string=hsa-miR-134-5p&search_type=mirna) \| 1207 \| [hsa-miR-25-3p](http://www.exiqon.com/ls/Pages/DeepLink.aspx?search_area=tpt&search_string=hsa-miR-25-3p&search_type=mirna) \| 567 \| |  |  |  |
| --- | --- | --- | --- | --- | --- | --- | --- | --- | --- | --- | --- | --- | --- | --- | --- | --- | --- | --- | --- | --- | --- | --- | --- | --- | --- | --- | --- | --- | --- | --- | --- | --- | --- | --- | --- | --- | --- | --- | --- | --- | --- | --- | --- | --- | --- | --- | --- | --- | --- | --- | --- | --- | --- | --- | --- | --- | --- | --- | --- | --- | --- | --- | --- | --- | --- | --- | --- | --- | --- | --- | --- | --- | --- | --- | --- | --- | --- | --- | --- | --- | --- | --- | --- | --- | --- | --- | --- | --- | --- | --- | --- | --- | --- | --- | --- | --- | --- | --- | --- | --- | --- | --- | --- | --- | --- | --- | --- | --- | --- | --- | --- | --- | --- | --- | --- | --- | --- | --- | --- | --- | --- | --- | --- | --- | --- | --- | --- | --- | --- | --- | --- | --- | --- | --- | --- | --- | --- | --- | --- | --- | --- | --- | --- | --- | --- | --- | --- | --- | --- | --- | --- | --- | --- | --- | --- | --- | --- | --- | --- | --- | --- | --- | --- | --- |


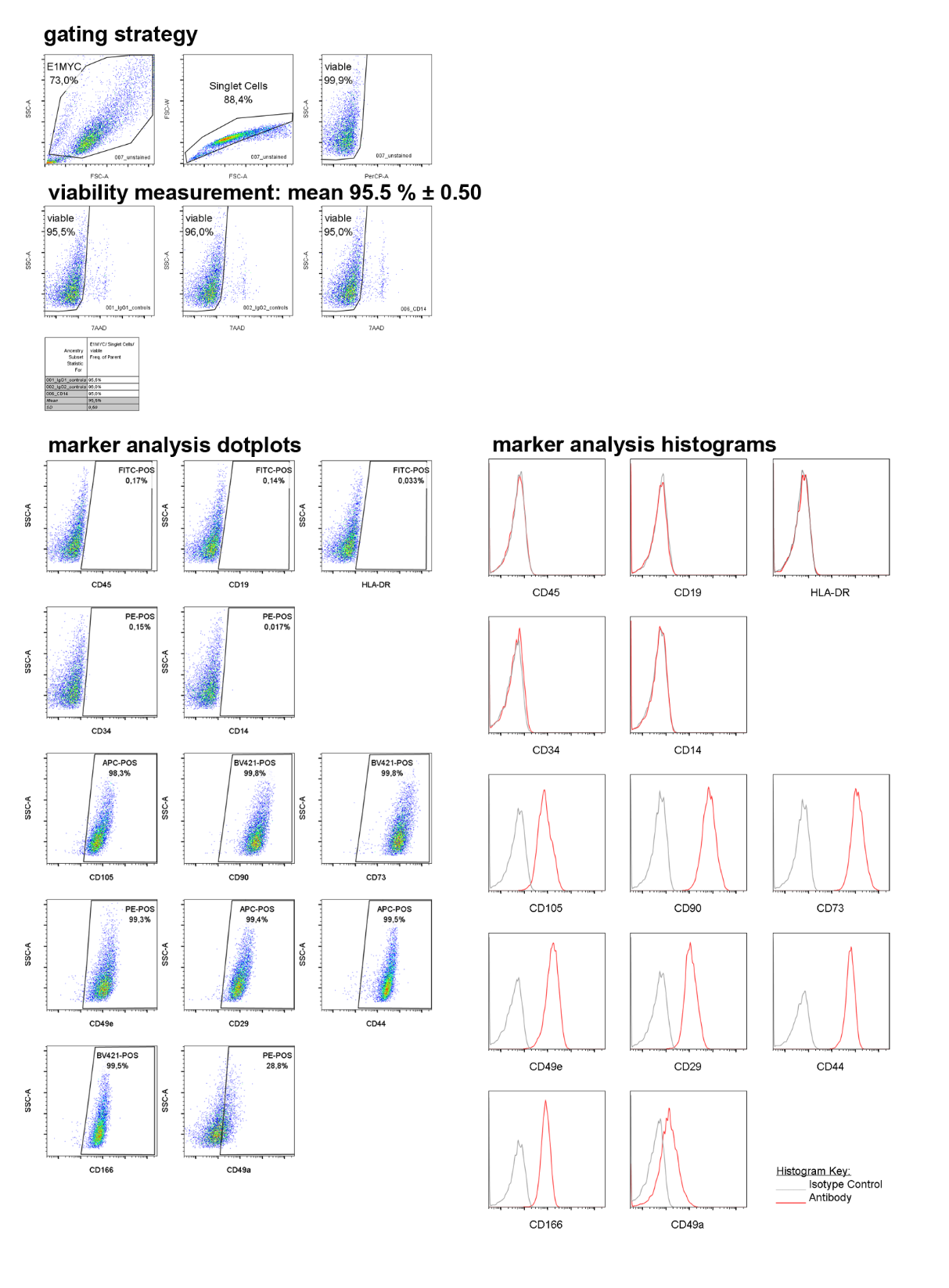


**Supplemental Figure S1** Immunophenotype and viability of producer cells for the UC-MSC-EV preparations. Gating strategy, dot plots and histogram plots of one representative UC-MSC product used for EV preparation. Immunophenotype and viability analysis confirmed the defined marker profile for MSC identity as published by the International Society of Cell Therapy (Dominici et al., 2006).


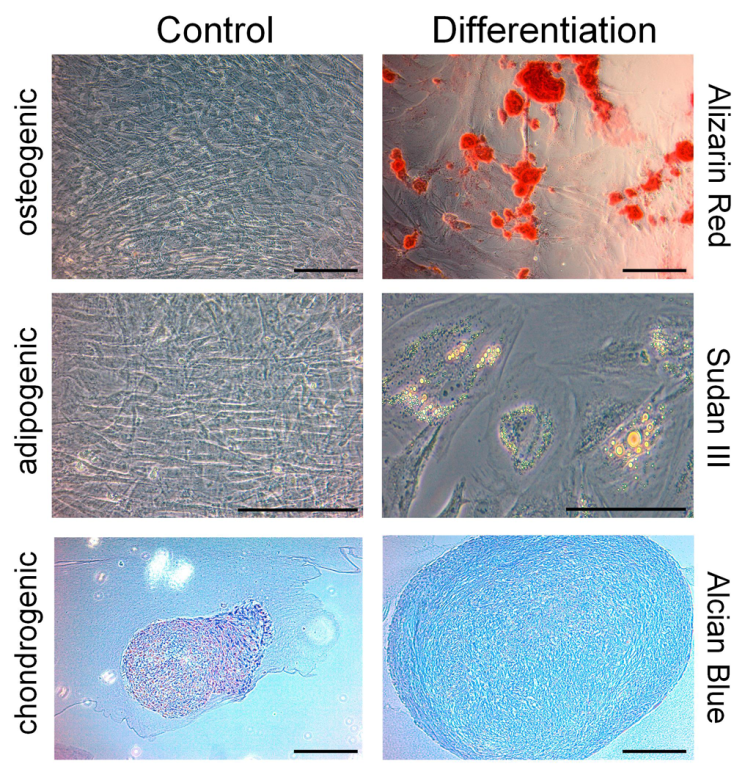


**Supplemental Figure S2** Tri-lineage differentiation of MSC. The MSC were incubated for 21 days either with osteogenic or adipogenic medium and were stained after fixation with Alizarin Red or Sudan III. Normal MSC medium served as control. For chondrogenic differentiation the MSCs were pelleted and incubated in chondrogenic medium containing TGF-$ß3.$ The staining for chondrogenic differentiation is Alcian Blue. The lineage specific staining demonstrated that the MSC used in this study were able to undergo a tri-lineage differentiation. Scale bar: 100 µm.

**Supplemental Figure S3** CD73 expression by clinical-grade UC-MSC-EVs. Flow cytometric MACSplex analysis of UC-MSC-EVs reveals the presence of CD73 on extracellular vesicles in addition to tetraspanins (CD9, CD63 and CD81) and typical MSC markers. The CD73 expression is normalized to the median intensity of the CD9, CD63, CD81-**BV421** signal


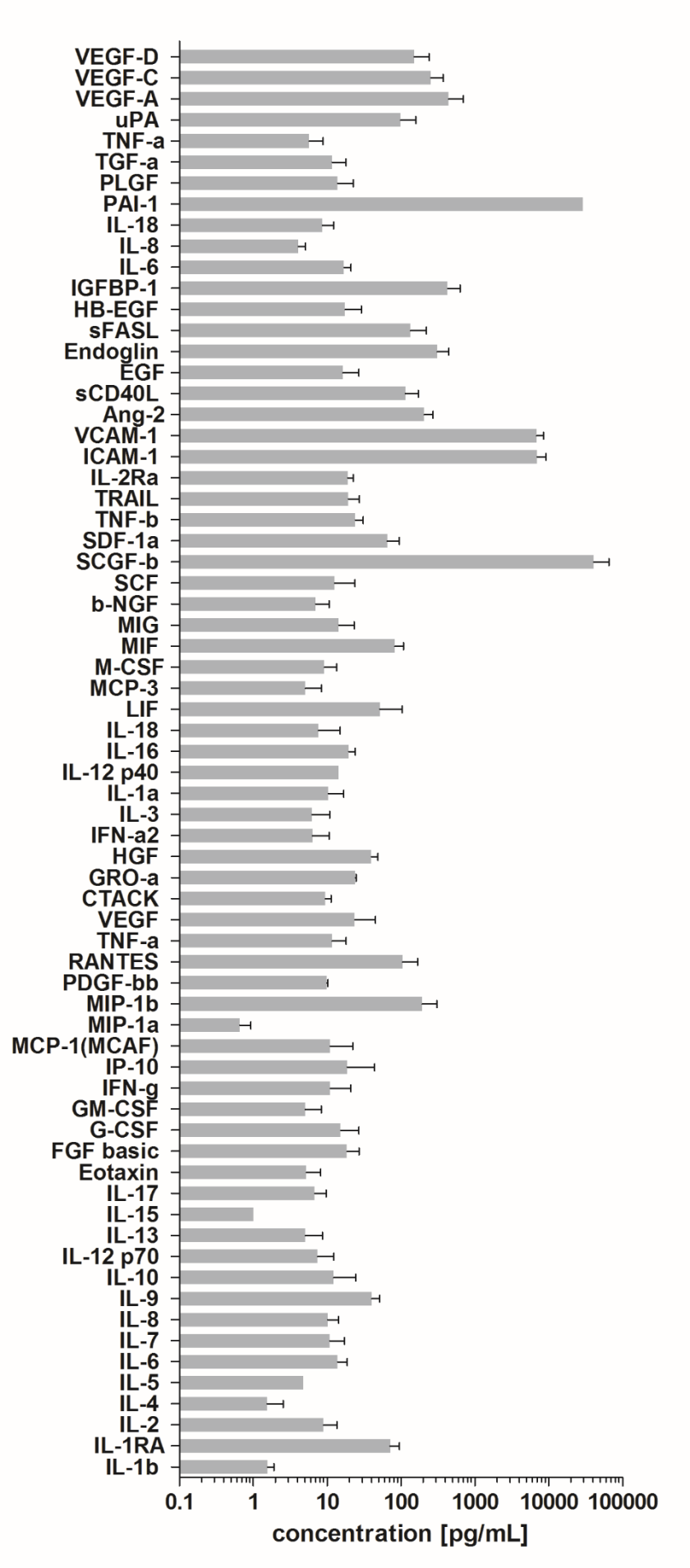


**Supplemental Figure S4** A Luminex-based multiplex array was applied to screen for a specific subset of human chemokines, cytokines and endothelial markers in MSC-EVs. The X-axes equal the logarithmic concentration scales in pg/mL. The samples were analysed in duplicates. All the factors that were included for analysis are depicted.


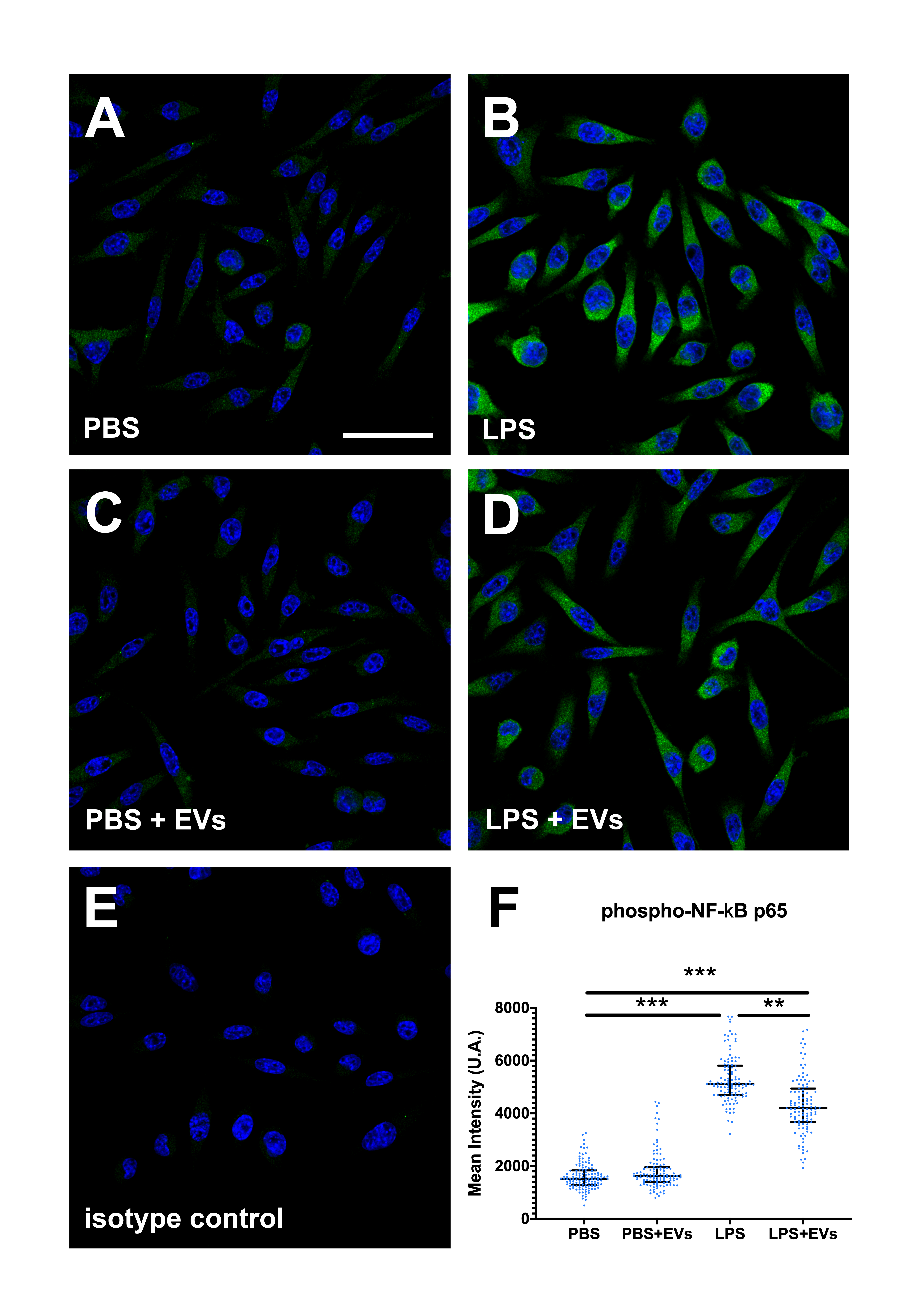


**Supplemental Figure S5** Analysis of NF-κβ p65 phosphorylation in BV-2 microglial cells. Immunodetection of phospho-NF-κβ p65 (green) in BV-2 cells 2 hours after application of (A) PBS, (B) 100 ng/mL LPS, (C) PBS and 1.2 x 10^8^ particles/mL and (D) 100 ng/mL LPS together with 1.2 x 10^8^ particles/mL. (E) isotype control using an unspecific rabbit primary antibody. DAPI nuclear counterstain (blue). Scale bar in (A) = 50 µm. (F) Quantification of phospho-p65 cytoplasmic signal intensity in the various conditions (arbitrary units, AU; mean ± standard deviation, **: p<0.01; ***: p<0.001).


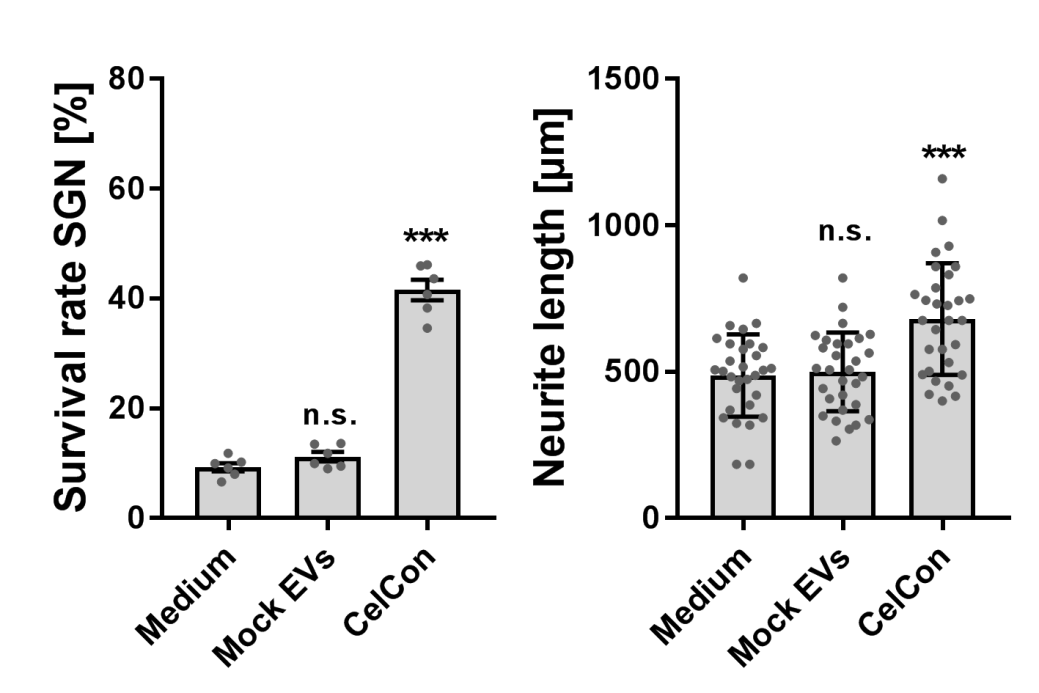


**Supplemental Figure S6** Treatment of SGN with mock EVs. The SGN were seeded in 50 µL of SGN medium. Then, a volume of 50 µL of the different factors was added per well: medium (SGN medium without any additional factors), Mock EVs (non-conditioned MSC medium submitted to TFF and ultracentrifugation, same treatment as the conditioned medium for EV isolation) and clinical grade EVs in a concentration of 2 x 10^6^. Treatment with the Mock EVs did not significantly increase the survival rate and the neurite length of SGN. The survival rate and neurite length were on a comparable level to the medium control. This clarifies that the neuroprotective effect did not derive from non-MSC-derived factors of the initial MSC medium. Number of experiments: N = 2; number of replicates n = 3. Each data point represents the survival rate (left) of a single well or the neurite length (right) of one measured neuron.

**References:**

Dominici, M., Le Blanc, K., Mueller, I., Slaper-Cortenbach, I., Marini, F., Krause, D., et al. (2006). Minimal criteria for defining multipotent mesenchymal stromal cells. The International Society for Cellular Therapy position statement. *Cytotherapy* 8, 315–7. doi:10.1080/14653240600855905.

Laner-Plamberger, S., Lener, T., Schmid, D., Streif, D. A., Salzer, T., Öller, M., et al. (2015). Mechanical fibrinogen-depletion supports heparin-free mesenchymal stem cell propagation in human platelet lysate. *J. Transl. Med.* 13, 354. doi:10.1186/s12967-015-0717-4.
